# MOBILE pipeline enables identification of context-specific networks and regulatory mechanisms

**DOI:** 10.1038/s41467-023-39729-2

**Published:** 2023-07-06

**Authors:** Cemal Erdem, Sean M. Gross, Laura M. Heiser, Marc R. Birtwistle

**Affiliations:** 1 Department of Chemical and Biomolecular Engineering, Clemson University, Clemson, SC USA; 2 Department of Biomedical Engineering, Oregon Health & Science University, Portland, OR USA; 3 Department of Bioengineering, Clemson University, Clemson, SC USA

**Keywords:** Systems biology, Data integration, Mechanisms of disease

## Abstract

Robust identification of context-specific network features that control cellular phenotypes remains a challenge. We here introduce MOBILE (Multi-Omics Binary Integration via Lasso Ensembles) to nominate molecular features associated with cellular phenotypes and pathways. First, we use MOBILE to nominate mechanisms of interferon-γ (IFNγ) regulated PD-L1 expression. Our analyses suggest that IFNγ-controlled PD-L1 expression involves *BST2*, *CLIC2*, *FAM83D*, *ACSL5*, and *HIST2H2AA3* genes, which were supported by prior literature. We also compare networks activated by related family members transforming growth factor-beta 1 (TGFβ1) and bone morphogenetic protein 2 (BMP2) and find that differences in ligand-induced changes in cell size and clustering properties are related to differences in laminin/collagen pathway activity. Finally, we demonstrate the broad applicability and adaptability of MOBILE by analyzing publicly available molecular datasets to investigate breast cancer subtype specific networks. Given the ever-growing availability of multi-omics datasets, we envision that MOBILE will be broadly useful for identification of context-specific molecular features and pathways.

## Abstract (web summary)

A problem in network biology is identification of context-specific networks. Here the authors report Multi-Omics Binary Integration via Lasso Ensembles (MOBILE) to nominate molecular features associated with cellular phenotypes and pathways, and use this to assess interferon-γ regulated PD-L1 expression.

## Introduction

The availability of large-scale multi-omics datasets across cell types, tissues, and organisms is rapidly increasing with the development of new technologies^1–7^. The challenge now is in analyzing them, not individually, but rather together to leverage the fact that signals are encoded across multiple modalities^8–11^. Examples of individual methods include: principal components analysis^12^, statistical approaches^13–15^, clustering^16–19^, unsupervised learning^20^, and supervised/machine learning^10,21–24^. Often, such analyses are used to generate networks where genes (or other biomolecules) are nodes, and edges between them denote statistical or functional relationships. Integrating data from multiple modalities can give rise to novel biological insights that cannot be obtained through the analysis of single datasets^25–27^. To date, data integration methods have produced systems-level biological insights, including genetic and protein–protein interactions^28–30^, disease-gene relationships^24,31^, drug response predictions^30,32^, and context-specific associations^29,31,33^.

A central problem in network biology is the identification of “context-specific” networks. Here we define context-specificity as a biological relationship (i.e., edge in a network) that applies only to a certain cell type, ligand perturbation, extracellular matrix component, or time point. Data integration has the potential to lead to significant advances in our understanding of such context-specific biology by identifying biological signals evident across multiple modalities^8^. For instance, why does insulin, but not insulin-like growth factor I (IGF1), regulate glucose metabolism despite their textbook signaling pathways being essentially identical^34–36^? Why does IGF1 activate ERK or AKT signaling only in certain cell types^37,38^? Identifying and studying context-specific observations is important because most diseases are tissue-specific^39^, and this knowledge can enable understanding of how different cell types respond to varied perturbations, which is key to targeted therapeutic intervention.

Multiple data integration methods have been implemented using bioinformatics tools, including kernels^40,41^, correlation analysis^42–45^, and others^46^. These methods were applied -but not limited- to processing genomic and epigenomic datasets to cluster and classify the input data. Published methods also include network (protein–protein or genomic interaction) inference using omics datasets, including Bayesian approaches^47,48^ and natural language processing^49^. One group of data integration approaches use prior literature/network knowledge as an essential first step^28–33,50–53^. For example, a prior knowledge-informed network analysis may limit exploration to curated pathways and remove analytes (measured features) with no known interactions, or may impose biological structure into the underlying network models^29,30^. These literature-driven approaches assemble available tissue-specific expression data into gene correlation networks^30,54,55^ or overlay the data on global (non-specific) interaction networks^9,28,56^. For instance, employing a network/graph theory-based approach coupled with prior literature information, differential network analysis^56–63^ tools showed promise in identifying context-specific knowledge and highlighted genes and pathways for clinical impact^11,59,64–69^. While informative, the drawback of such “literature-first” data integration methods is that they only allow the study of known connections, which omits the substantial number of regulatory interactions not annotated in the literature^30,56,70^. Importantly, these approaches cannot identify novel interactions or associations in the datasets.

Another group of data integration approaches are prior knowledge agnostic, which circumvents the limitations of literature-first methods^24,44,71–73^. These methods rely on extracting features from the input data and constructing models to discriminate between conditions. For instance, Zhang et al. used deep learning to integrate gene expression and copy number variance data from two different databases to identify distinct prognostic subtypes^24^. However, the trade-off of these methods is that they are more challenging to interpret because they lack direct incorporation of a biological structure (e.g., central dogma) into the data integration methodology and also do not take advantage of the wealth of available prior knowledge^11,74,75^.

Despite progress, there remains a need for new tools and methods for biologically informed multi-omics integration without literature-driven pre-selection. Here, we introduce **M**ulti-**O**mics **B**inary **I**ntegration via **L**asso **E**nsembles (MOBILE) to integrate multi-omic datasets and identify context-specific interactions and pathways. Our approach does not eliminate data based on prior knowledge and also uses a well-established central dogma structure to aid biological interpretation. Robust associations are inferred between pairs of chromatin accessibility regions, mRNA expressions, and protein/phosphoprotein levels. By imposing this high-level structure, MOBILE is neither network structure agnostic (for better interpretability) nor heavily prior knowledge bound (for higher rates of novelty). We demonstrate the method using a recent multi-omic dataset generated by the NIH LINCS Consortium^76^. In that project, non-tumorigenic breast epithelial MCF10A cells were assayed for proteomic, transcriptomic, epigenomic, and phenotypic changes in response to six growth factor perturbations (EGF, HGF, OSM, IFNγ, TGFβ1, and BMP2; synapse.org/LINCS_MCF10A). We apply MOBILE to this dataset and obtain candidate context-specific associations. We then use these associations (i) to propose sub-networks of regulation for therapeutically important genes and (ii) to identify pathways preferentially activated by pairs of ligands from similar signaling families. First, MOBILE identifies regulatory mechanisms for IFNγ-controlled PD-L1 expression that have independent literature support. Secondly, MOBILE reveals–and independent experiments validate–that TGFβ1 but not BMP2 induces laminin pathway genes (especially laminin 5), causing stronger cell-to-cell and cell-to-surface adhesion through interactions with extracellular collagen, which leads to larger and more separated cells. Finally, we use MOBILE to explore breast cancer subtype-specific pathways by integrating patient-level transcriptomic and proteomic datasets from The Cancer Genome Atlas (TCGA)^1^. The biologically structured and data-driven MOBILE pipeline outlined here is widely applicable to integrate omics datasets for extracting context-specific network features and insights.

## Results

### A multi-omics LINCS perturbation dataset for integrative analysis

The NIH LINCS Consortium recently released a unique and comprehensive multi-omics dataset (synapse.org/LINCS_MCF10A) that consists of molecular and phenotypic responses of MCF10A cells to multiple ligand perturbations over time^76^. Spanning a compendium of canonical receptor signaling classes, EGF, HGF, and OSM induced growth, while BMP2, IFNγ, and TGFβ1 inhibited growth. The cellular responses were measured using live-cell imaging, immunofluorescence (IF), and cyclic immunofluorescence^77^. The bulk molecular responses were assessed across five platforms. The proteomic assay was reverse phase protein array (RPPA^78^), where specific phospho- or total protein levels were measured at 1, 4, 8, 24, and 48 h. The RNAseq transcriptomic dataset was single-end sequencing at 24 and 48 h. Chromatin accessibility was profiled by Assay for Transposase-Accessible Chromatin using sequencing (ATACseq), also at 24 and 48 h after stimulation. A pretreatment (T0 control) was quantified for all assay types. Overall, the MCF10A dataset provides an excellent template for applying the proposed data integration strategies, namely ATACseq, RNAseq, and RPPA as “big data” to be integrated, and the live-cell imaging / IF as assays informing associated cellular phenotypes.

### The MOBILE integrator

**Fig. 1:**
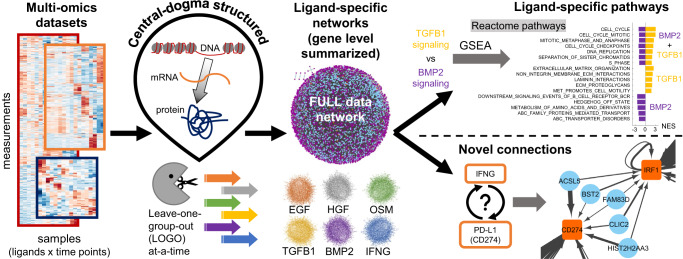
Multi-Omics Binary Integration via Lasso Ensembles (MOBILE) pipeline yields statistically robust, ligand-specific association networks. The MOBILE data integrator combines multi-omics, multi-assay datasets in a data-driven and central-dogmatic way. By leaving each ligand condition out from the input at a-time, the pipeline outputs robust ligand-specific association networks. These gene-level networks are used to infer differentially enriched pathways and to find regulatory sub-networks.

**Fig. 2:**
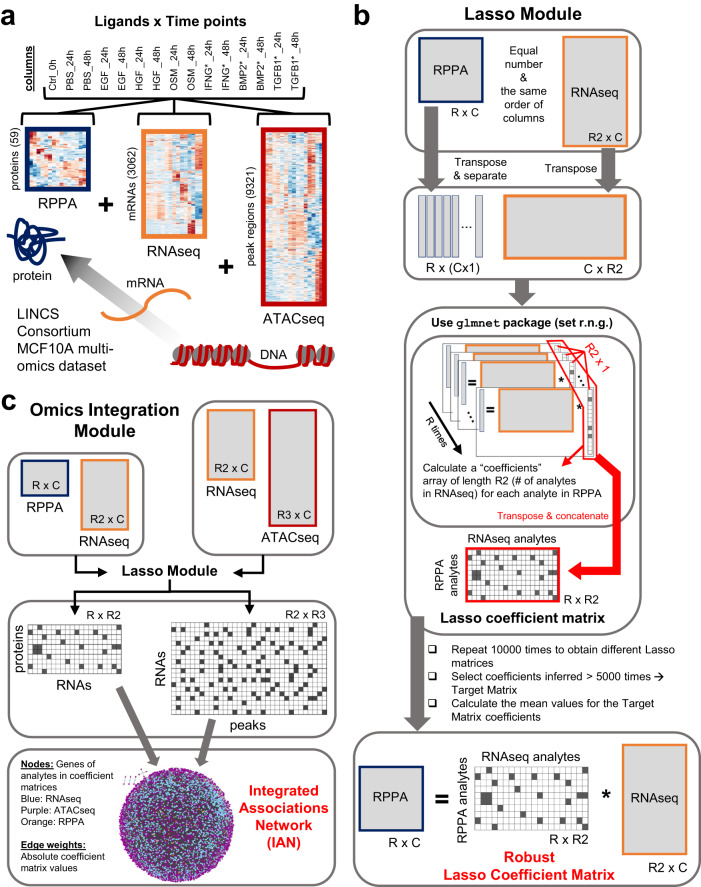
The MOBILE Integrator pipeline transforms input data into gene-level association networks. **a** The LINCS MCF10A datasets include proteomic (RPPA), transcriptomic (RNAseq), and epigenomic (ATACseq) assays. The number of analytes retained after data preprocessing are shown in parentheses on the y-axis. The heatmaps shown are the results from the hierarchical clustering of rows. Source data are provided as a Source Data file. **b** The Lasso module is used to integrate omics datasets one pair at a time. The associations between chromatin peaks (ATACseq) and mRNA levels (RNAseq) and between mRNA levels and protein levels (RPPA) are calculated separately. The two assay input matrices are structured to yield a Lasso coefficient matrix, which contains association coefficients between analytes of the two input matrices. Ten thousand instances of the Lasso matrices are generated. The coefficients that appear in at least half the matrices (>5000 times) are considered robust and the mean values of these coefficients populate the Robust Lasso Coefficient Matrix (RLCM). The non-zero elements in this matrix are called associations for the remainder of this work. **c** The Robust Lasso Coefficient Matrices of two input pairs are combined to generate Integrated Association Networks (IANs). These gene-level networks represent robustly, statistical associations inferred from multi-omics datasets, offering a new hypotheses generation tool to look for ligand or gene-set specific sub-networks. Node colors represent; blue:RNAseq, purple:ATACseq, and orange:RPPA, and the edge widths correlate with the magnitude of the association coefficients.

**Fig. 3:**
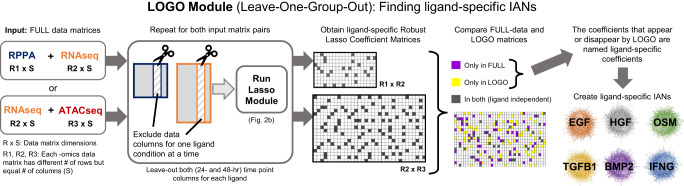
The leave-one-group-out (LOGO) module generates ligand-specific association networks. We employed a leave-one-group-out (LOGO) analysis to find associations associated with exclusion/inclusion of that specific condition. We excluded one set of ligand conditions (24 and 48 h) from the input matrices, ran the Lasso module (Fig. 2b) with a smaller number of columns, and obtained a new Robust Lasso Coefficient Matrix, named ligand-specific coefficient matrices. Comparing the resulting matrix from the LOGO module to the FULL-data Lasso coefficient matrix, we determined the “ligand-dependent” coefficients (ones that appear only in LOGO IAN and coefficients that are present only in FULL-data IAN) to create the final ligand-specific associations list. Repeating the process for each ligand treatment condition, we obtained six ligand-LOGO IANs.

We here integrated data from this LINCS dataset to identify context-specific pathways and regulatory mechanisms that control cellular phenotypes. The overall approach is summarized in Fig. 1 and presented in greater detail in Figs. 2, 3. The availability of epigenomic, transcriptomic, and proteomic datasets inspired a central-dogmatic view for data integration (Fig. 1). For compatibility across datasets and operability of the method, the MOBILE pipeline input included all three datasets with all ligands at 24 and 48 h only.

Following the central dogma that information flows from DNA to RNA to protein, we paired ATACseq-RNAseq and RNAseq-RPPA matrices. First, we calculated robust and parsimonious statistical associations between features of input data (Fig. 2a, see Methods) using replicated penalized regression models (Fig. 2b and Supplementary Data 1,2). We applied Lasso (least absolute shrinkage and selection operator^37,79^) regression to infer sets of sparse matrices that can be interpreted as statistical networks connecting biochemical species, such as mRNAs, chromatin peaks, or total protein and phosphorylation levels. The repetitive application of Lasso, called the Lasso module, yielded an ensemble of matrices, from which we picked one as the robust associations matrix. When we used all the ligand conditions from the LINCS dataset as input, the Lasso module output is called the FULL-data matrix. To finalize the data integration and generate data-driven networks, we merged the robust associations matrices obtained from RPPA+RNAseq and RNAseq+ATACseq input pairs into an Integrated Association Network (IAN) (Fig. 2c). The IANs are coalesced gene-level networks, where nodes represent genes of the assay analytes (genes from input matrix rows) and edges represent robust Lasso coefficients calculated between the analyte levels (Supplementary Fig. 1).

We then systematically excluded different ligand conditions (both 24- and 48-h data) from the training input and ran the LOGO (leave-one-group-out) module (Fig. 3). The input data with a smaller number of columns were then processed via a regular Lasso module (Fig. 2b, c), and new Robust Lasso Coefficient Matrices were generated. Next, by comparing the FULL-data associations matrix to the newly inferred LOGO-condition associations matrix, we identified associations dependent on the exclusion/inclusion of that specific condition’s data. This “ligand”-specific association list is used to create the ligand-LOGO-IAN. Repeating the process for each ligand condition in the dataset, we obtained six ligand-specific IANs (Fig. 3).

We hypothesized that the robust associations that change as a result of this LOGO analysis will have information regarding the context-specificity of the left-out ligand condition. The ligand-specific IANs, together with the FULL IAN were the major data-integration products of the MOBILE. Comparison of pairs of ligand IANs nominated differentially activated networks and ligand-specific regulatory mechanisms spanning DNA states to protein levels. To facilitate biological interpretation, we performed gene-set enrichment analysis (GSEA^80^) using the Reactome database^81^ that enabled us to identify pathways linked to ligand-specific IANs. We then nominated ligand-specific pathways and novel edges associated with distinct phenotypes observed in the image data.

### MOBILE identifies known biology

**Fig. 4:**
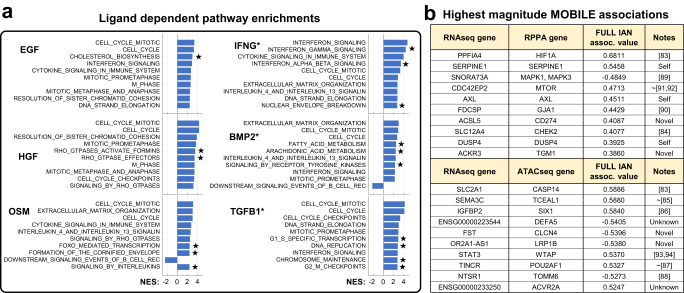
MOBILE inferred IANs are enriched for canonical pathways and the top associations are literature-verified interactions. **a** Gene set enrichment analysis using genes of ligand-dependent associations revealed cell cycle, interferon, and cytokine signaling pathways. Only the top ten significantly enriched pathways are shown for each ligand perturbation (*p* values <0.05, FDR <0.1, significance tested using default GSEA settings of single-tail null distributions^80^). The bar height corresponds to the normalized enrichment scores (NES). Stars denote pathways enriched specifically for the corresponding ligand condition when only considering the top ten pathways shown. Asterisk denotes conditions with additional EGF treatment. Source data are provided as a Source Data file. **b** MOBILE finds known and novel associations between proteins, transcripts, and chromosomal region genes. The top ten magnitude-wise associations between RPPA-RNAseq and RNAseq-ATACseq of the FULL IAN are presented. More than half of the associations have prior literature evidence, while some are “Self” associations of the same gene in different assays. Two associations include non-annotated gene products labeled as “Unknown”. At least four of the associations are “Novel” predictions of the MOBILE pipeline. ~ denotes references not showing a direct (causative) relationship between the genes but co-mentioning them as biomarkers of different cancer subtypes or with relationships of candidates’ isoforms. The MOBILE inferred association values are between −1 and 1.

We investigated the robustness of the MOBILE predictions by performing a gene-set enrichment analysis (GSEA) for pathways using the FULL and ligand-specific integrated associations networks (IANs) and asking whether our approach can capture canonical biological observations. First, we identified ligand-dependent association lists by comparing each ligand IAN to the FULL IAN. Next, these association lists were coalesced into gene-level networks and the nodes were ranked based on the sum of edge weights (association magnitudes) of that node (Supplementary Data 3). We ran GSEA on these eight (FULL, PBS, EGF, HGF, OSM, IFNG*, BMP2*, and TGFB1*) pre-ranked gene-lists and found that the top enriched pathways were cell cycle in all conditions (*p* < 0.05 and FDR <0.1, Fig. 4a and Supplementary Data 4). Indeed, eight of the top 15 pathway enrichments across conditions are cell cycle-related, affirming the fact that the LINCS dataset was generated using combinations of pro/anti-growth factors and cells continue to grow after all perturbations (Supplementary Fig. 2 and Supplementary Data 5).

Other highly enriched pathways in the HGF dependent gene-list were Rho GTPase related (Fig. 4a). It was shown before that Rho GTPase activity is required for HGF-induced cell scattering^82^. OSM-dependent pathway enrichments included cytokine/interleukin and ECM pathways (Fig. 4a). The top four highly enriched pathways of IFNG* condition were interferon signaling, in line with the fact that IFNγ had a strong signal in the LINCS dataset^76^. BMP2-dependent top pathways were ECM, interferon, and interleukin related in addition to the cell cycle. Finally, the TGFB1* condition had transcriptional and DNA regulatory pathways enriched (Fig. 4a). These observations confirmed that our approach can recover known biology and also that it can extract meaningful ligand-specific associations.

Next, we asked whether MOBILE-inferred associations (edges) are consistent with prior knowledge. The highest magnitude associations of the FULL analysis are the most robust across all perturbations and time points (Fig. 4b, Supplementary Data 1,2). Among them, the top candidate interaction is the connection between *PPFIA4* (Protein Tyrosine Phosphatase Receptor Type F Polypeptide-Interacting Protein Alpha-4) and *HIF1A* (Hypoxia Inducible Factor 1 Subunit Alpha). The *PPFIA4* gene was shown to be upregulated in response to hypoxia (through *HIF1*) in all types of breast cancer cell lines and normal-like epithelial cells, including MCF10A^83^. The highest association between ATACseq and RNAseq data is the *SLC2A1* (Solute Carrier Family 2 Member 1) and *CASP14* (Caspase 14). Interestingly, these two genes were also part of the hypoxia-induced genes list^83^. There exists literature evidence for the other highest-ranking associations (Fig. 4b). Some were (i) shown to be part of prognostic markers (*SLC12A4*-*CHEK2*^84^), (ii) differentially expressed together in response to perturbations (*SEMA3C*-*TCEAL1*^85^, *IGFBP2*-*SIX1*^86^, *TINCR*-*POU2AF1*^87^, *NTSR1*-*TOMM6*^88^), and (iii) part of gene signatures for different classes of tumors (*SNORA73A*-*MAPK*^89^, *FDCSP*-*GJA1*^90^). A few of the associations had related mechanistic interactions as well *(CDC42-MTOR*^91,92^, *IGFBP2-SIX1*^86^*, WTAP-STAT3*^93,94^*, TINCR-POU2AF1*^87^). The remaining associations are either Self: same gene, different data type, Unknown: non-curated gene(s), or Novel: no known interactions to the best of our knowledge. These pieces of information from the literature, in part, verify that the MOBILE inferred associations have biological meaning.

### Identification of associations between IFNγ stimulation and PD-L1 regulation

**Fig. 5:**
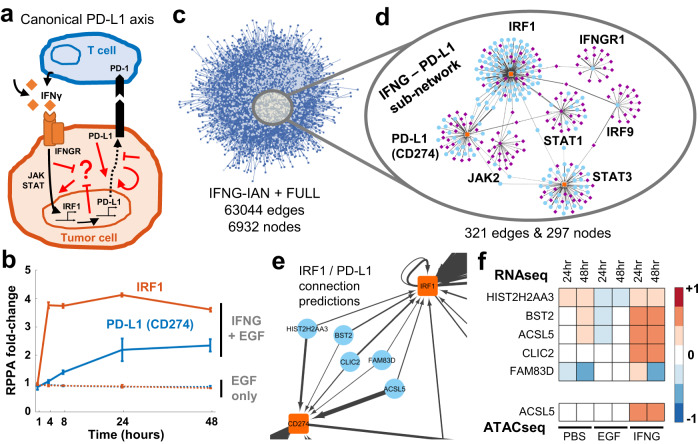
Exploration of a single integrated association network reveals new links between IFNγ signaling and PD-L1. **a** IFNγ secreted by T cells induces PD-L1 expression through JAK/STAT/IRF1 and other canonical pathways (black arrows). The PD-L1 on the cell surface then interacts with PD-1 on the immune cells to induce tumor cell death. However, the PD-1/PD-L1 therapy yields inter- and intratumor heterogeneous responses, and there is a need to identify new non-canonical targets (represented by red arrows). **b** The IFNγ induces IRF1 and PD-L1 production in MCF10A cells. Data shown are from three independent biological replicates and error bars represent the standard error of the mean. Source data are provided as a Source Data file. **c** The IFNG associations' network (IFNγ-IAN) is a data-driven large-scale network of connections. The associations are coalesced into gene-level nodes, and associations with greater than 0.01 absolute value are shown. **d** The sub-network of the IFNγ – PD-L1 relationship is significantly smaller than the IFNγ-IAN. The sub-network is generated by filtering for 14 genes and has seven hubs (PD-L1 (*CD274*), *IFNGR1*, *IRF1*, *IRF9*, *JAK2*, *STAT1*, *STAT3*) connected to 290 other genes. Node colors represent; blue:RNAseq, purple:ATACseq, and orange:RPPA and the edge widths correlate with the magnitude of the association coefficients. **e** A closer look at the connections between *IRF1* and PD-L1 (*CD274*) genes shows a breadth of different functional genes. **f** Compared to no or EGF-only stimulation, the connecter genes are upregulated (RNAseq) in IFNγ stimulated condition. Additionally, the *ACSL5* peak is more accessible similar to the canonical IFNγ downstream genes (ATACseq). The data shown are the average values from three independent biological replicates. Source data are provided as a Source Data file.

After establishing that MOBILE can recapitulate known biological interactions, we asked whether it could identify regulatory mechanisms within a single IAN. We focused on IFNγ, which had a strong signal in the LINCS dataset^76^ and is a critical part of the immune response within the tumor microenvironment^95,96^. The cytokines within the environment, especially IFNγ, can induce transient PD-L1 (gene name: *CD274*) expression (Fig. 5a)^97–100^. PD-L1 is a transmembrane protein that binds to its receptor PD-1 expressed in T cells and inhibits immunological tumor clearance. Both PD-L1 and PD-1 belong to a class of so-called “checkpoint” proteins;^98,101^ immune checkpoint inhibitors are a new class of immunotherapeutic anti-cancer drugs^100,102^. However, PD-L1 expression is not correlated with patient response and is highly variable depending on tumor stage, site, and type. Consequently, predicting tumor responses to PD-1/PD-L1 blockade remains a challenge, and better biomarkers are needed to stratify patients. Therefore, an in-depth understanding of the regulatory mechanism of PD-L1 expression is still needed to provide new immunotherapeutic insights and potentially identify new drugs^103^. To investigate this question, we decided to explore sub-networks between IFNγ signaling and PD-L1 expression within the data-driven IFNγ integrated associations network.

In the LINCS dataset, IFNγ was tested in combination with EGF, so we isolated the IFNγ response by comparing the IFNγ condition (IFNγ + EGF) to EGF-only samples. We confirmed that IFNγ stimulation uniquely upregulated canonical downstream elements, including IRF1, interferon regulatory factor, and PD-L1 (*CD274* gene) (Fig. 5b). Next, we identified a set of nine genes (*IFNG*, *IFNGR1*, *IFNGR2*, *STAT1*, *STAT3*, *JAK1*, *JAK2*, *IRF1*, and *IRF9*) from the canonical IFNγ pathway (REACTOME R-HSA-877300) and filtered the connections of the genes together with PD-L1 (*CD274*) and PD-1 (*CD279*) from IFNG-IAN (Fig. 5c). The resulting sub-network had 297 nodes and 321 edges (Fig. 5d and Supplementary Data 6,7). The hubs in that sub-network were from the input gene-list, including *IRF1*, *STAT1*, *STAT3*, and *CD274* (PD-L1). We examined the connections between IRF1 and PD-L1 (Fig. 5e) and identified five nodes: *BST2*, *CLIC2*, *FAM83D*, *ACSL5*, and *HIST2H2AA3*. The mRNA levels of these five genes were elevated in the IFNγ + EGF condition compared to EGF-only samples (Fig. 5f). Of the five genes, three had strong literature support. *BST2* was recently shown to be part of a gene signature for anti-CTLA4 response in melanoma^104^. *CLIC2* is co-expressed with PD-L1/PD-1 in breast cancer and is a biomarker candidate for favorable prognosis^105^. And although not directly linked to IFNγ/PD-L1 axis, *FAM83D* was shown to regulate cell growth and proliferation and was implicated as a prognostic marker in breast and gastric cancers^106–108^. Moreover, its *FAM83A* isoform was shown to affect PD-L1 expression^109^. We could not find any literature data for two genes (*ACSL5* and *HIST2H2AA3*) and their relationships to IFNγ and PD-L1 function, suggesting potentially novel diagnostic or therapeutic targets for immunotherapy. Importantly, the concordance of some findings with recent literature reinforces the notion that MOBILE-based nomination of interactions has biological value.

### TGF-β superfamily members TGFβ1 and BMP2 induce different morphological phenotypes via collagen-laminin signaling

Both BMP2 and TGFβ1 are members of the TGF-β superfamily and share most downstream pathways, including canonical SMAD signaling^110,111^. Both ligands induce cell differentiation and show anti-growth/anti-proliferative effects, whereas SMAD signaling shows immense versatility and specificity, mostly affected by the cross-talk mechanisms and the cellular context^110–118^.

**Fig. 6:**
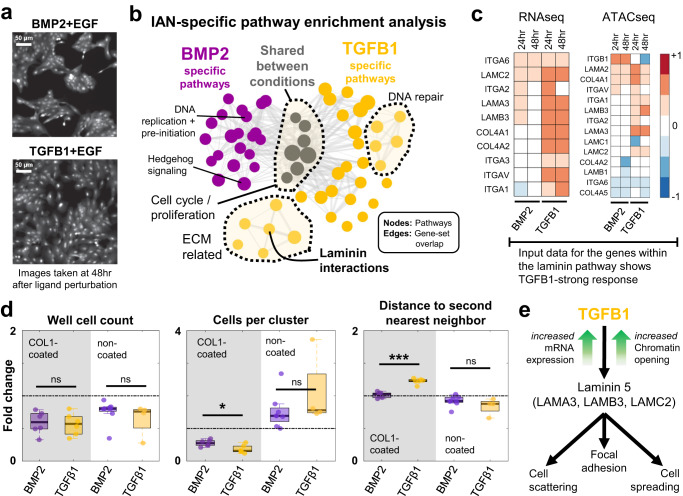
TGFβ1 and BMP2 induce different morphological phenotypes by differentially activating collagen-laminin signaling. **a** TGFβ1 and BMP2 induce phenotypically different responses at 48 h after ligand treatment^76^. Representative images are adapted examples from eight biological replicates. **b** Both TGFβ1 and BMP2 networks are enriched in cell cycle and proliferation-related pathways (gray circles). TGFβ1 alone is shown to regulate ECM-related pathways, whereas BMP2 network genes are linked to other membrane receptor-related signaling pathways and cell cycle checkpoints. Node size is proportional to the enrichment scores and edge widths represent the number of overlapping genes. **c** The expression levels of the laminin pathway genes (left) are upregulated by TGFβ1. The chromosomal regions (peaks, right panel) of the laminin genes are more accessible in the TGFβ1 condition. Transcriptomic and epigenomic data shown are the average values from three independent biological replicates. Source data are provided as Source Data file. **d** BMP2 (purple) and TGFβ1 (gold) induce similar anti-growth responses but yield different microenvironmental and spatial characteristics. Both ligands inhibit cell proliferation (well cell counts, shaded region). BMP2 induces a larger number of cells in clusters compared to TGFβ1 (cells per cluster, shaded region, *p* value = 0.0175). TGFβ1 causes cells to spread and have a longer distance to their second nearest neighbors (distance to second neighbors, shaded region, *p* value = 9.4032E-06). Cells grown in non-collagen-coated regular tissue-culture plates show phenotype reversal of cells per cluster and distance-to-second-neighbor metrics (unshaded regions, middle and right box plots), with no significant cell number differences between BMP2 and TGFβ1. The box edges correspond to the 25th−75th percentiles, the horizontal black lines represent the median, and the dots are individual data points. The whiskers extend to the non-outlier extremes. ns: not significant, asterisk: *p* value <0.05, three asterisks: *p* value <0.001. Significance using two-sided Student’s *t*-test with unequal variance. Data shown are from *N* = 6, 6, 8, and 4 biological replicates respectively. Source data are provided as Source Data file. **e** The schematic of TGFβ1 specific regulation of a non-SMAD pathway, inferred by the MOBILE pipeline. TGFβ1 induces laminin pathway gene expression and leads to cell scattering and cell spreading with larger cells, when compared to BMP2 stimulation.

Imaging data of cells grown on collagen-coated culture plates from LINCS^76^ indicated that BMP2 induces a significantly higher number of cells in clusters as compared to that induced by TGFβ1 (Fig. 6a, top). Correspondingly, TGFβ1 induced morphologically larger cells^119,120^ that occupy more surface area (Fig. 6a, bottom). We used the ligand-specific IANs (Supplementary Fig. 3) and subsequent pathway enrichment analyses to find non-canonical mechanisms that underly the differential phenotypes caused by these two highly similar ligands. We ranked the nodes of the TGFβ1 and BMP2 IANs (Supplementary Data 8,9) based on the sum of edge weights entering that node (Supplementary Data 10, 11) and ran GSEA on these pre-ranked gene-lists^80,121^. We then looked for curated Reactome pathways^81^ significantly enriched (*p* < 0.05 and FDR <0.1) in either gene-list (TGFβ1 or BMP2 IAN genes) (Fig. 6b). Seven of the enriched pathways under BMP2 and TGFβ1 treatment conditions are shared (Fig. 6c gray circles, and Supplementary Data 12,13). The shared pathways are all cell cycle and proliferation-related. Multiple pathways are specific to a single condition (Fig. 6b, BMP2: purple and TGFβ1: gold). BMP2-enriched pathways include DNA regulation and G1/S transition. The TGFβ1-only group has DNA damage and ECM regulation-related clusters of pathways. It is important to note that pathway enrichment analyses on shuffled data-derived TGFβ1 and BMP2-specific IANs did not yield any pathways (*p* value <0.05, and FDR <0.1).

The laminin interactions (REACTOME R-HSA-3000157) is among the TGFβ1-only enriched pathways. Laminins are a family of proteins that regulate cell-to-cell and cell-to-matrix interactions^122–127^, by binding to collagen^128^ and integrin receptors^129^. Depletion of ECM laminin or collagen disrupts cellular attachment^122,130^. Differential regulation of these processes might offer a candidate mechanism for the observed TGFβ1 and BMP2 phenotypic difference: only TGFβ1 induces laminin gene expression that then interacts with the collagen-coating of the culture plate, which induces tighter cell-to-cell and cell-to-ECM interactions leading to more spreading and stretching cells. So, we analyzed the activity of the laminin pathway under TGFβ1 and BMP2 stimulation by comparing the mRNA levels (the MOBILE input data) of the pathway genes (Fig. 6c). The mRNA levels of laminin, collagen, and integrin subunits were elevated at 24 and 48 h (Fig. 6c, left), and corresponding transcription binding sites were more accessible in TGFβ1 stimulated cells (Fig. 6c, ATACseq data, right).

In the LINCS dataset, cells were cultured and treated on collagen-coated plates^76^ and both ligands inhibited cell proliferation as compared to EGF-only control (Fig. 6d, left panel). Additionally, BMP2 induced more cell clustering compared to TGFβ1 (Fig. 6d, middle panel), but TGFβ1 was shown to cause significantly larger distances to second nearest neighbors (Fig. 6d, right panel). To test the hypothesis that TGFβ1-specific phenotypes depend on activation of laminin-collagen (in ECM) interactions (Fig. 6e), we cultured cells on non-collagen-coated plates and stimulated them with TGFβ1 or BMP2 (+EGF, as done in the LINCS dataset^76^). Removal of the collagen caused a reversal of the TGFβ1-induced phenotype. The number of cells per cluster and distance to the second nearest neighbors are similar in BMP2 and TGFβ1 stimulated cells in the absence of collagen-coating (Fig. 6d, middle and right panels). Overall, these analyses confirmed that MOBILE identified a context-specific network that explains differential phenotype between two highly similar ligands.

Finally, although MOBILE analysis identified the laminin pathway as potentially explanatory for differential phenotype, we wondered whether such a conclusion could be reached by standard differential expression analysis. Considering the same list of pre-filtered 3062 transcripts that were input to the MOBILE pipeline, we determined BMP2 or TGFβ1 up and downregulated genes at 24- or 48-h conditions. Next, we ranked the genes based on the fold-change (BMP2 vs TGFβ1 or TGFβ1 vs BMP2) and, similar to post-MOBILE enrichment analysis, we ran GSEA on these pre-ranked lists of genes (Supplementary Data 14). We then compared MOBILE results with the differential expression analysis results. The BMP2 upregulated, TGFβ1 upregulated, BMP2 downregulated, and TGFβ1 downregulated gene-lists alone did not yield any significant pathway enrichments (*p* value <0.05 and FDR <0.1). However, we obtained a single significantly enriched pathway (REACTOME Extracellular Matrix Organization, R-HSA-1474244) when we looked at the combined list of TGFβ1 up- and down-regulated genes. The combined BMP2 regulated gene-list yielded 10 significantly enriched pathways, including ECM Organization (Supplementary Data 15), whereas the MOBILE pipeline yielded 39 (TGFβ1, Supplementary Data 12) and 20 (BMP2, Supplementary Data 13) enriched pathways using TGFβ1- and BMP2-specific IANs. This indicates that the MOBILE inferred ligand-specific association networks and their analyses extract more information about differential pathway enrichments as compared to standard methods.

### MOBILE infers subtype-specific and differentially activated pathways using paired-omics datasets from TCGA samples

MOBILE is agnostic to data sources and can be applied to a variety of datasets when structured correctly. We show the applicability of the pipeline by using tumor transcriptomic (RNAseq) and proteomic (RPPA) data from the TCGA database^1^. In short, 878 samples were identified to have both transcript and protein data from the primary breast tumor site. Analysis of receptor expression was used to stratify these samples into clinically-relevant subtypes: 89 HER2-amplified (HER2-amp), 129 triple-negative (TNBC), and 308 estrogen and progesterone receptor positive (ER+/PR+) (see Methods and Supplementary Fig. 4).

**Fig. 7:**
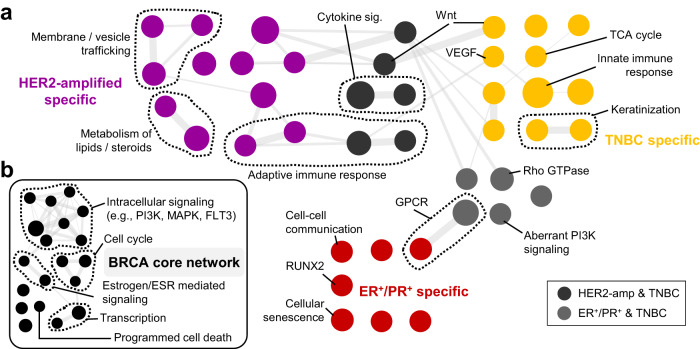
MOBILE infers breast cancer subtype-specific and differentially activated pathways. **a** The LOGO-IANs are pair-wise compared and subtype-specific pathway enrichments are determined. The colors represent subtypes (purple: HER2-amplified, gold: TNBC, red: ER/PR-positive, gray: subtype shared), node size depends on pathway gene-set size, and edge width correlates with shared gene-set similarity of the connected nodes. Examples of pathways are labeled individually or as groups. **b** The pathways enriched in all LOGO-IAN conditions constitute the breast cancer (BRCA) core network. The list includes cell cycle, estrogen, MAPK, and PI3K signaling as well as transcriptional regulation and programmed cell death. Node size and edge width represent the gene-set size and gene-set similarity of the connected nodes, respectively.

Following the MOBILE Lasso Module procedure (Fig. 2b) and using all samples (878 cases), we generated a FULL breast cancer integrated association network (FULL-TCGA-IAN). Next, we calculated subtype-specific IANs with the LOGO module (Fig. 3). Then, the subtype IANs are pair-wise compared to each other and enriched Reactome pathways are determined (Fig. 7a, Supplementary Fig. 5, and Supplementary Data 16–18). Specifically, the HER2-amplified subtype was enriched in membrane trafficking, lipid/steroid metabolism, and adaptive immune response-related pathways (Fig. 7a, purple and dark gray). In support of these observations, the literature data shows that HER2-amplified cell lines and tumor samples both have elevated FASN and ACAT1 function (fatty acid synthesis), leading to poor prognosis^131–134^ and inhibitors to such targets enhance the efficacy of anti-HER2 therapies^135^. HER2-amplified cells were also shown to have high levels of endocytic and extracellular vesicle trafficking^136^.

TNBC is a subtype with poor prognosis and aggressive phenotype. The MOBILE inferred TNBC-specific enriched pathways include receptor signaling pathways like Wnt and VEGF, keratinization, TCA cycle, and innate immune response-related pathways (Fig. 7a, gold and dark gray). In the literature, the TNBC subtype was shown to correlate with high VEGF activity and multiple antiangiogenic agents were included in combination therapies for aggressive tumors like TNBC^137,138^. Similarly, elevated and dysregulated Wnt signaling in TNBC was reported numerously^139,140^. In recent studies, suppression of the TCA cycle was shown effective in decreasing the growth and invasion of TNBC cells^141^, as increased TCA cycle utilization provides elevated ATP production for the cells^142,143^.

Finally, the ER+/PR+ subtype was enriched in GPCR and aberrant PI3K signaling (together with TNBC), cell-cell communication, cellular senescence, and RUNX2-mediated transcription pathways (Fig. 7a, red and light gray). In the hormone receptor-positive subtype, hyper-activated PI3K activity was previously shown^144–147^. Moreover, cell-cell communication and signaling is elevated ER+ cell lines^148^, where chemotherapy-induced senescence is sensitive to hormone therapy^149^. Regarding RUNX2-mediated transcriptional activity, triple-positivity (ER+, PR+, RUNX2+) is prevalent, especially in grade 2 and 3 breast tumors^150^, with possible ER-RUNX2 mutual regulatory mechanisms^151^. Finally, G protein–coupled receptor superfamily, and especially the G-protein-coupled estrogen receptor (GPER)^152,153^, was shown to mediate estrogen-dependent kinase activity in breast cancer tumorigenesis and metastasis^154–156^.

In addition to the subtype-specific enriched pathways, we summarized the common pathways enriched in all three subtypes (Fig. 7b and Supplementary Data 19). These include cell cycle, transcription, intracellular signaling, and programmed cell death pathways. Estrogen receptor (ESR)-mediated signaling was also included in the core network, most likely due to the facts that estrogen is critical and central in breast biology and the curated pathway gene-list coincides largely with major proliferation pathways (MAPK and PI3K) and transcription.

## Discussion

Here we introduced the MOBILE pipeline to integrate and analyze multi-omics datasets in a data-driven way. Using a central-dogmatic framework, the method finds statistically robust integrated association networks (IANs) between pairs of epigenomic, transcriptomic, and proteomic analytes that are biologically interpretable. We explored context-specific IANs obtained via leave-one-group-out (LOGO) analysis, yielding (i) non-canonical connections with novel immunotherapeutic potential, (ii) differentially activated pathways to discriminate between highly similar ligand-receptor responses, and (iii) breast cancer subtype-specific pathway enrichments.

The MOBILE pipeline can prioritize context-specific, differentially activated pathways and mechanisms. MOBILE identifies statistically significant associations, while LOGO analysis provides context-specificity. In our analysis of the LINCS MCF10A dataset, by holding out data from each ligand one at a time, associations that depend on the held-out data are inferred and cataloged as ligand-dependent. These ligand-dependent associations are the core of MOBILE integrator, enabling the exploration of single ligand-specific and differentially enriched pathways between multiple conditions. We expect that the exact method used for forming statistical associations (replicated Lasso) could be substituted with other methods^10,24,28,30,54–56^. However, by imposing matching time points, we miss time-lagged associations between mRNAs and proteins due to their temporal ordering. Thus, the next step for the MOBILE pipeline could be to become more flexible with respect to different time points for different assays to more fully exploit temporal dependence information for inference of associations.

The above-mentioned matching column order for input matrices requirement of the MOBILE pipeline in this work does not restrict users to study only time points x ligand conditions. For instance, we applied MOBILE to TCGA datasets to infer cancer subtype-specific pathways, where the columns of input matrices were individual patients. Moreover, we previously showed that paired input data matrix pairs at different time points (rows: proteomic measurements, columns: different cell lines) could infer robust, time-dependent associations between proteins and phospho-proteins^37^. Another way to utilize the MOBILE pipeline is to find cell line-specific association networks by considering datasets like the Cancer Cell Line Encyclopedia (CCLE), where hundreds of cell lines were characterized with molecular and functional assays^2,157^. MOBILE could be set up where columns represent different cell lines, and the matrix pair are proteomic and transcriptomic data. By imposing different higher-level hierarchies for the MOBILE pipeline, researchers can explore different types of context-specificity by using data from either single or multiple assays.

The current MOBILE pipeline is appropriate for use with continuous data only. This is partly due to the datasets we analyzed so far and in part due to the Lasso regression type selected. However, other methods within the glmnet package could be substituted, which would provide additional flexibility on the input data. Another limitation of the current framework is that input matrices need to be complete, without missing values; future versions of MOBILE could include data imputation methods to further enhance functionality and enable the use of sparse data.

Nevertheless, the associations generated by MOBILE are all data-driven experimental candidates to study ligand-specific linkages between genes and gene products. The highest magnitude associations could suggest new hypotheses and inform the design of experiments to explore cross-talk mechanisms or unknown links in the literature. For instance, by analyzing the IFNγ-specific network only, we hypothesized new regulatory mechanisms of PD-L1, a critical immunotherapeutic target. Of the five MOBILE-hypothesized connector genes (*BST2*, *CLIC2*, *FAM83D*, *ACSL5*, and *HIST2H2AA3*) between IRF1 and PD-L1, *BST2* was recently recognized as part of an immune/tumor-related signature that is significantly associated with the overall survival of skin cancer patients^104^. Specifically, the *BST2* gene signature predicted a response to a CTLA4 antibody called ipilimumab, suggesting a mechanistic involvement in tumor progression. Secondly, *CLIC2* was shown to be co-expressed with PD-L1 and PD-1 and act as a good prognosis marker with higher rates of tumor-infiltrating CD8 + T cells in breast cancer patients^105^. Finally, *FAM83D* was shown to be a potential oncogene with high expression levels associated with poor breast cancer prognosis^106^. Moreover, *FAM83A* (an isoform of *FAM83D*) was shown to drive PD-L1 expression and be correlated with poor lung cancer prognosis^109^. These results suggest that context-specific gene-gene associations identified through MOBILE are potential biomarkers for prognosis and patient response to immune checkpoint inhibition.

Another key capability of the MOBILE pipeline is the IAN-comparative analysis. For instance, we hypothesized and then experimentally validated that the phenotypic differences between TGFβ1 and BMP2 perturbations are caused by cell-ECM interactions, specifically laminin-collagen. When we assessed responses in the absence of collagen-coating, the two ligands induced similar changes, in agreement with prior findings that both TGFβ1 and BMP2 induce cell differentiation, inhibit cell proliferation, and signal through similar canonical pathways^111–113^. Additionally, it was shown before that laminin/collagen pathway inhibition leads to cell-ECM attachment disruption^122,130^. However, there are other TGFβ1-specific ECM-related pathways (Fig. 6b) and genes that could be further explored for differences between BMP2 and TGFβ1 conditions. Similarly, studying other ligand-IAN pairs (e.g., EGF vs HGF, IFNγ vs TGFβ1, and OSM vs IFNγ) could suggest additional data-driven hypotheses.

We also demonstrated that MOBILE performs well with patient data. By empirically defining three patient subtypes and applying LOGO, we determined association networks for HER2-amplified, triple-negative, and ER+/PR+ subtypes. The pair-wise comparison of corresponding IANs revealed subtype-specific and subtype-independent (core) enriched pathways. Most of the pathways were previously reported in association with the mentioned subtype, but MOBILE also provided hypotheses to explore further. An example of the latter is that the HER2-amplified subtype network was enriched for the adaptive immune response pathway, whereas the TNBC subtype was associated with innate immune response and keratinization pathways. Another next step would be to explore patient-specific IANs using the same TCGA input.

The MOBILE pipeline here infers robust associations between genes and gene products without prior network knowledge input, enables the generation of context-specific, gene-level networks of different biological modalities in a data-driven way, and provides an exploration of these networks in a single or paired fashion to pinpoint differentially activated pathways. We believe the freely-available MOBILE pipeline will be broadly helpful in extracting context-specific insights from multi-omics datasets to help answer targeted biological questions.

## Methods

### The Lasso module

The multi-omics datasets from the LINCS consortium^76^ are pre-processed using a raw variance filter to retain only 10% (RNAseq, ATACseq) and 20% (RPPA) highly variant analyte measurements across median summarized Level 4 data (Fig. 2a, synapse.org/LINCS_MCF10A, 10.6084/m9.figshare.20294229.v2). The variance cut-off provides that we only look at analytes with some variability across different (ligand and time point) conditions^158–160^. The difference in cutoff percentages is due to the difference in number of features across assays. RPPA had the least, thus retained at a higher percentage (20 compared to 10). The proteomic (RPPA), transcriptomic (RNAseq), and epigenetic (ATACseq) datasets are integrated with a central-dogmatic view, such that pairs of RPPA+RNAseq and RNAseq+ATACseq data matrices are run through the Lasso module (Fig. 2b, c). The steps of the algorithm^37^ are:

1. We use glmnet package for lasso regression^161^. The module takes two input matrices, **Y**=left hand side and **X**=right hand side, and our goal is to calculate matrix **β** in ${{{{{\bf{Y}}}}}}{{{{{\boldsymbol{=}}}}}}{{{{{\boldsymbol{\beta }}}}}}\cdot {{{{{\bf{X}}}}}}{{{{{\boldsymbol{+}}}}}}{{{{{\boldsymbol{\delta }}}}}}$. The number and the ordering of columns in input matrices should be equal (Fig. 2b). The rows are assay analytes measured and columns represent ligand/time point conditions.
2. The matrices are column-centered, row-centered, and row normalized. The preprocessing makes sure that the algorithm is not biased towards high-magnitude analyte measurements but focused on analyzing based on the shape of measurements across conditions. It also sustains that the offset value is moved toward zero.
3. We set the cross-validation parameter of glmnet package to 4 and turned off the input data standardization option.
4. Next, both matrices are transposed and the transposed **Y** matrix (**Y’**) is separated into column vectors.
5. For each column **k** of the **Y’** (or each row of input matrix **Y**), a set of lasso regression coefficients are calculated using glmnet package. With every iteration, we obtain one row of the final coefficient matrix **β** and an offset value *δ*, which is negligible in this case (values less than 10^−7^). We minimize the quantity:
  1 $${\sum }_{i=1}^{C}{\left[{Y}_{{ki}}-{\sum }_{j=1}^{R}{\beta }_{{kj}}{X}_{{ji}}-{\delta }_{k}\right]}^{2}+\lambda {\sum }_{j=1}^{R}\left|{\beta }_{{kj}}\right|$$
6. The λ factor (Eq. 1) is estimated via the inherent cross-validation step of the glmnet package. In short, a set of different λ values are tested, resulting in different sets of lasso coefficients, each with a potentially different number of non-zero coefficients.
7. We select the set of lasso coefficients (i.e., the lasso coefficient vector) corresponding to the minimum estimation error.
8. Repeating steps 5-7 as many times as the number of input **Y** rows, we obtain R-many Lasso coefficient vectors, each R2-long.
9. We concatenate the R-many R2-long vectors to obtain a Lasso coefficient matrix.
10. We repeat the (3–9) steps 10,000 times to obtain an ensemble of Lasso coefficient matrices. We start each estimation with a different seed for a random number generator.
11. We calculate the average value for every matrix position, based only on the matrices that are non-zero. We also find the coefficient indices (matrix positions) that appear (inferred as non-zero) at least half of the time (currently ≥5000 times). The overlap between these two matrices is called the Robust Lasso Coefficient Matrix (RLCM) and used for the rest of the analyses.
12. The final matrix (**β**) sustains the equality ${{{{{\bf{Y}}}}}}={{{{{\boldsymbol{\beta }}}}}}\cdot {{{{{\bf{X}}}}}}$ and contains association weights relating to the analyte levels of the two input matrices.
13. When the input data contains all experimental conditions, we named the resulting robust Lasso coefficient matrix as the FULL-data matrix.
14. We do steps 1-13 for (i) RPPA (matrix **Y**)-RNAseq (matrix **X**) and (ii) RNAseq (matrix **Y**)-ATACseq (matrix **X**) input data matrix pairs.
15. To show that the inferred coefficients are non-random, we repeated the above steps for sets of shuffled input matrices, using Matlab (R2018a and R2021b) randperm function. We saw that the randomized input matrices resulted in a significantly smaller number of coefficients inferred (Supplementary Fig. 6). We used the kstest2 function in Matlab to test for the significance in the differences (Kolmogorov-Smirnov distances) between real and shuffled conditions. We obtained *p* values = 0 for all comparisons, indicating that the real input has more information content and thus requires more coefficients to explain the data.

The MOBILE simulation of RPPA-RNAseq inference takes around 2–3 s per run (10,000 instances are run in total: ~8 h), including the save function in a normal desktop/laptop. The RNAseq-ATACseq inference simulations were run on Clemson University Palmetto HPC and took around 8 h per 1000 iteration of the 10,000 instances (sources used per batch job: number of nodes = 1, number of CPUs = 40, memory = 360 gb). Ten batch jobs were run in parallel, and the results were concatenated offline afterward.

### The LOGO module

In addition to the Lasso module, we employ the leave-one-group-out (LOGO) module (Fig. 3) to obtain a new robust Lasso coefficient matrix for each perturbation in the input dataset. Here, the perturbations are ligand combinations used. We create a ligand-specific matrix by excluding that ligand condition during the model run and comparing the resulting matrix to FULL-data robust Lasso coefficient matrix to determine the coefficients that depend on the existence of the corresponding ligand data. We apply the LOGO module for both RPPA-RNAseq and RNAseq-ATACseq input pairs. Similar in principle to cross-validation, the LOGO module here enabled us not just to integrate given datasets but to acquire ligand-specific associations.

### The integration

When both proteomic-transcriptomic and transcriptomic-epigenetic robust Lasso coefficient matrices are obtained, they are merged into a single, gene-level network (Fig. 2c). This network representation is named the Integrated Associations Network (IAN), where each node is a gene, and edge weights represent the Lasso coefficient magnitudes. Notably, the gene nodes can represent data from one or more RPPA, RNAseq, or ATACseq sets. Summarizing the networks at the gene level enabled us to explore pathway enrichments using GSEA^80^.

### GSEA and pathway enrichments

Using the Lasso+LOGO modules and excluding one ligand condition at a time, we obtain seven LOGO IANs (PBS, EGF, HGF, OSM, IFNγ, TGFβ1, and BMP2) in addition to the FULL-data network. We compare each ligand network with the FULL-data network to determine ligand-dependent associations and create gene-level network visuals using Cytoscape^162^. Next, we calculate the weighted sum of edge weights for each gene node in the networks and rank them. Then, we run pathway enrichment analysis using GSEA^80^ and Reactome to obtain a list of curated pathways enriched for each network (i.e., ligand-LOGO condition).

We calculate the gene-level weight α for each gene k (Eq. 2) by summing over each edge width and normalizing by the total number of possible edges.

2 $${\alpha }_{{{gene}}_{k}}=\frac{{\left|{\sum }_{j=1}^{3062}{\sum }_{i=1}^{59}{\beta }_{{ij}}\right|}_{{RPPA}{{\_}}{RNAseq}}+{\left|{\sum }_{j=1}^{3062}{\sum }_{i=1}^{9321}{\beta }_{{ij}}\right|}_{{RNAseq}{{\_}}{ATACseq}}}{59\cdot 3062\cdot 9321}$$

The ranked gene-lists are then imported into GSEA software (version 4.1.0) and used in the GSEAPreranked analysis. We select Reactome (v7.2) as the gene set database, keep the default 1000 permutations option, and keep the dataset as is without collapsing the gene symbols since we already use the HGNC identifiers. We also choose the classical weighting option and choose “149” as our seed for permutation for reproducibility. We repeat these steps for every ligand-specific ranked gene-list from the ligand-specific IANs.

After the enrichment analysis successfully completes, we use the Enrichment Map Visualization tool of GSEA, together with Cytoscape (v3.7.1). We only retain pathways enriched with a *p* value less than 0.05 and a false discovery rate (FDR) of 0.1. The results for BMP2 and TGFβ1 are given in Supplementary Data 12, 13. For this comparison, we only retain coefficients with magnitudes larger than 0.1 (Supplementary Fig. 7).

### MOBILE performance on finding known interactions

We determined the lists of genes represented in the RPPA-RNAseq and RNAseq-ATACseq input matrices and used them to obtain known (literature-based) interactions via stringApp^163^ in Cytoscape^162^ (Supplementary Data 20, 21). Then, we counted the overlap between the literature interaction lists and 10,000 Lasso coefficient matrices of real (yellow distributions) and shuffled (orange and blue distributions) data (Supplementary Fig. 8).

The real data derived Lasso matrices contained significantly more interactions “correctly” inferred in both RPPA-RNAseq and RNAseq-ATACseq cases. For the latter, the real data condition yielded twice the number of known interactions in post-selection shuffled data and almost twenty times the number of interactions inferred from pre-selection shuffled data. The number of known interactions identified by the Robust Lasso Coefficient Matrices are also counted and reported (dashed lines, Supplementary Fig. 8).

### TCGA data analysis

TCGA breast cancer transcriptomic (RNAseq) and proteomic (RPPA) datasets are downloaded (accessed on November 12, 2022, Supplementary Data 22). A total of 1226 RNAseq and 919 RPPA data files were obtained, representing 1098 cases (or patients). The data are filtered for paired files per case with the primary tumor site only. The final list was comprised of 878 cases (data columns). Then, the datasets are further cleaned: (i) ribosomal nuclear, long noncoding, micro, and other unannotated RNA transcripts and transcripts measured only in less than 10% cases are excluded from RNAseq and (ii) measurements with NaN values in RPPA are removed. The finalized lists for the MOBILE pipeline contained 27,797 transcript levels (RNAseq) and 457 protein levels (RPPA).

Next, three subtypes are determined using RNAseq measurements: (i) HER2-amplified (log2(HER2_fpkm+1) > 7), (ii) triple-negative (TNBC, log2(HER2_fpkm+1) < 7, log2(ER_fpkm+1) < 1, and log2(PR_fpkm+1) < 1), and (iii) estrogen and progesterone receptor positive (ER+/PR+, log2(HER2_fpkm+1) < 7, log2(ER_fpkm+1) > 3, and log2(PR_fpkm+1) > 3). About 89, 129, and 308 cases were selected for each subtype, respectively. Then, following MOBILE pipeline guidelines, only the top highly variant analytes (10% for RNAseq, 20% for RPPA) are retained. MOBILE Lasso Module (Fig. 2b) is run using all samples (878 columns) to obtain the FULL-TCGA-IAN. By excluding sample columns for each subtype from the input data, we ran the LOGO module (Fig. 3) to obtain context-specific IANs. Finally, the subtype-LOGO-IANs are pair-wise compared to each other, and enriched pathways are determined.

### Cell culture

MCF10A cells (ATCC #CRL-10317, acquired from LINCS Consortium/Gordon Mills and STR verified internally in March 2019) are cultured in DMEM/F12 (Gibco #11330032) medium supplemented with 5% (by volume) horse serum (Gibco #16050122), 20 ng/mL EGF (PeproTech #AF-100-15), 0.5 mg/mL hydrocortisone (Sigma #H-0888), 10 μg/mL insulin (Sigma #I-1882), 100 ng/mL cholera toxin (Sigma #C-8052), and 2mM l-Glutamine (Corning #25-005-CI). Cells were cultured at 37 ^o^C in 5% CO_2_ in a humidified incubator and passaged every 2–3 days with 0.25% trypsin (Corning #25-053-CI) to maintain subconfluency. Experimental starvation medium is DMEM/F12 medium supplemented with 5% (by volume) horse serum (Gibco #16050122), 0.5 mg/mL hydrocortisone (Sigma #H-0888), 100 ng/mL cholera toxin (Sigma #C-8052), and 2 mM l-glutamine (Corning #25-005-CI).

### Mycoplasma testing

The cells were tested for Mycoplasma using a detection kit (Lonza #LT07-701). Following the manufacturer protocol, growth media (2 mL) from the culture plate was spun at 200×*g* for 5 min, and 100 μL from the cleared supernatant was transferred into a well of a 96-well plate. About 100 μL testing Reagent was added into the same well and let settle for 5 min. Then luminescence is measured in Synergy H1 microplate reader (Agilent Technologies, Inc., CA, USA) with Gen5 (v3.08) software. The Gain was set to 200, the integration time to 1 s, and a single reading was captured. Then, the plate is returned under the hood and 100 μL of testing Substrate was added into the same well. The plate was left to settle for 10 min at room temperature. Then, the second luminescence reading (cell supernatant, Reagent, and Substrate) was taken with the same parameters. A ratio of less than 1 indicates a negative test.

### Validation experiments

The cells were seeded in full growth media at 2000 cells/well in tissue culture treated (no collagen-coating) 96-well plates (Falcon #353072). After loading, plates were left to settle under the hood for 30 min and then placed in the incubator for 10 h. Next, the media is exchanged for experimental starvation media for 15 h. Then, media are replaced with fresh, experimental media containing the ligand(s): EGF (10 ng/mL, R&D Systems #236-EG), BMP2 (20 ng/ml, R&D Systems #355-BM) + EGF (10 ng/ml), and TGFβ1 (10 ng/ml, R&D Systems #240-B) + EGF (10 ng/ml). Each condition was repeated in triplicate. The plates are incubated for ~48 h. The cells are fixed using %2 paraformaldehyde (Alfa Aesar #43368) and stained with Hoechst (1:10000, by volume, BD Biosciences #561908) for nucleus localization. The plate was left for 1 h at room temperature. The wells are washed once with 1X PBS and replenished with 40 μl/well PBS.

### Imaging

The plates are imaged at 10X magnification with phase contrast objective (Agilent/BioTek part number 1320516) and TagBFP filer cube (Agilent/BioTek part number 1225115, excitation 390 nm, emission 447 nm). A total of 10 × 8 fields of view per well? are imaged with laser autofocus on the Cytation5 (Agilent Technologies, Inc., CA, USA). When reading was done, the tiles were montaged together by the Gen5 (v3.04, Agilen/BioTeK) software using phase contrast images as registration template (fusion method = linear blend, final image reduction to 13.71%). The imaging parameters for phase contrast were LED = 10, Integration time = 8, and Gain = 24. The parameters for the TagBFP channel were LED = 10, Integration time = 36, and Gain = 24.

### Image processing and quantification

The images are processed for cell segmentation and finding cell centroids. In short, TrackMate (v7.1.0) plugin from ImageJ (v2.3.0/1.53 f) is used to locate cell nuclei in each image (Hoechst stain + BFP channel, see 10.6084/m9.figshare.20294229.v2) and the summary was exported as a comma separated file (see 10.6084/m9.figshare.20294229.v2). The parameters for the plugin were Detector=LoG, Estimated object diameter=5 pixels, Quality threshold=0, Pre-process with median filter=ON, Sub-pixel localization=ON, and Initial thresholding=Auto.

### Spatial and microenvironmental metric calculations

The csv files exported by ImageJ included each cell object as a row and reported its center coordinates with other default information. The files were imported, and the cells per cluster and distance-to-neighbors metrics were evaluated using R (v4.1.3) scripts (on figshare 10.6084/m9.figshare.20294229.v2, github.com/cerdem12/MOBILE) and packages (readr v2.1.2, dplyr v1.0.9, ggplot2 v3.3.5, tidyr v1.2.0, stringr v1.4.0, LPCM v0.46-7, foreach v.1.5.2) and RStudio (2022.02.0 + 443 Prairie Trillium release). The script is adapted from github.com/MEP-LINCS/MDD/blob/master/R/MDD_Immunofluorescence_Lvl0Data_Processing.R^76^.

### Statistics and reproducibility

Although no statistical method was used to predetermine sample sizes, community standards were followed, and at least three biological replicates were run. The experiments were not randomized.

## .Supplementary information

Supplementary Information

Supplementary Inventory

Editorial Assessment Report

Supplementary Data 1

Supplementary Data 2

Supplementary Data 3

Supplementary Data 4

Supplementary Data 5

Supplementary Data 6

Supplementary Data 7

Supplementary Data 8

Supplementary Data 9

Supplementary Data 10

Supplementary Data 11

Supplementary Data 12

Supplementary Data 13

Supplementary Data 14

Supplementary Data 15

Supplementary Data 16

Supplementary Data 17

Supplementary Data 18

Supplementary Data 19

Supplementary Data 20

Supplementary Data 21

Supplementary Data 22

## Data Availability

The LINCS datasets analyzed during the current study are available in the Synapse repository, synapse.org/LINCS_MCF10A^76,164^. The data used to plot figure panels are provided in Source Data. The immunofluorescence data generated, processed LINCS data, and processed TCGA data are provided on figshare at 10.6084/m9.figshare.20294229.v2^165^. Source data are provided with this paper.

## Source data

Source Data

## Code availability

The final model scripts, files, and information are available at github.com/cerdem12/MOBILE^166^.

## References

[1] Koboldt DC (2012). Comprehensive molecular portraits of human breast tumours. Nature.

[2] Nusinow DP (2020). Quantitative proteomics of the cancer cell line encyclopedia. Cell.

[3] Barrett T (2012). NCBI GEO: archive for functional genomics data sets—update. Nucleic Acids Res..

[4] Edwards NJ (2015). The CPTAC data portal: a resource for cancer proteomics research. J. Proteome Res..

[5] Stathias V (2020). LINCS Data Portal 2.0: next generation access point for perturbation-response signatures. Nucleic Acids Res..

[6] Thul PJ (2017). A subcellular map of the human proteome. Science.

[7] Davis CA (2018). The encyclopedia of DNA elements (ENCODE): data portal update. Nucleic Acids Res..

[8] Dolinski, K. & Troyanskaya O. G. Implications of Big Data for cell biology. *Mol. Biol. Cell.* **26**, 2575–2578 (2015). 10.1091/mbc.E13-12-0756 PMC4501356 26174066

[9] Yao V, Wong AK, Troyanskaya OG (2018). Enabling precision medicine through integrative network models. J. Mol. Biol..

[10] Martorell-Marugán, J. et al. In *Computational Biology* (ed. Husi, H.) Ch. 3 (Codon Publications, 2019). 31815381

[11] Sealfon RSG, Wong AK, Troyanskaya OG (2021). Machine learning methods to model multicellular complexity and tissue specificity. Nat. Rev. Mater..

[12] Park M, Kim D, Moon K, Park T (2020). Integrative analysis of multi-omics data based on blockwise sparse principal components. Int. J. Mol. Sci..

[13] Jensen KJ, Janes KA (2012). Modeling the latent dimensions of multivariate signaling datasets. Phys. Biol..

[14] Kreeger PK (2013). Using partial least squares regression to analyze cellular response data. Sci. Signal.

[15] Lê Cao, K. A., Rossouw, D., Robert-Granié, C. & Besse, P. A sparse PLS for variable selection when integrating omics data. *Stat. Appl. Genet. Mol. Biol.* **7**, 35 (2008). 10.2202/1544-6115.1390 19049491

[16] de Souto MC, Costa IG, de Araujo DS, Ludermir TB, Schliep A (2008). Clustering cancer gene expression data: a comparative study. BMC Bioinformatics.

[17] Wiwie C, Baumbach J, Röttger R (2015). Comparing the performance of biomedical clustering methods. Nat. Methods.

[18] Oyelade, J. et al. Clustering algorithms: their application to gene expression data. *Bioinforma. Biol. Insights* **10**, 237–253 (2016). 10.4137/BBI.S38316 PMC5135122 27932867

[19] Abu-Jamous B, Kelly S (2018). Clust: automatic extraction of optimal co-expressed gene clusters from gene expression data. Genome Biol..

[20] Liu W, Payne SH, Ma S, Fenyö D (2019). Extracting pathway-level signatures from proteogenomic data in breast cancer using independent component analysis. Mol. Cell Proteom..

[21] Shipp MA (2002). Diffuse large B-cell lymphoma outcome prediction by gene-expression profiling and supervised machine learning. Nat. Med..

[22] Van’t Veer (2002). Gene expression profiling predicts clinical outcome of breast cancer. Nature.

[23] Soinov LA, Krestyaninova MA, Brazma A (2003). Towards reconstruction of gene networks from expression data by supervised learning. Genome Biol..

[24] Zhang L (2018). Deep learning-based multi-omics data integration reveals two prognostic subtypes in high-risk neuroblastoma. Front. Genet..

[25] Choi H, Pavelka N (2011). When one and one gives more than two: challenges and opportunities of integrative omics. Front. Genet..

[26] Buescher JM, Driggers EM (2016). Integration of omics: more than the sum of its parts. Cancer Metab..

[27] Huang S, Chaudhary K, Garmire LX (2017). More is better: recent progress in multi-omics data integration methods. Front. Genet..

[28] Oh M, Park S, Kim S, Chae H (2021). Machine learning-based analysis of multi-omics data on the cloud for investigating gene regulations. Brief. Bioinform..

[29] Kawata K (2018). Trans-omic analysis reveals selective responses to induced and basal insulin across signaling, transcriptional, and metabolomic networks. iScience.

[30] Dugourd A., et al. Causal integration of multi‐omics data with prior knowledge to generate mechanistic hypotheses. *Mol. Syst. Biol*. **17**, e97302021 (2021). https://onlinelibrary.wiley.com/doi/10.15252/msb.20209730. 10.15252/msb.20209730 PMC7838823 33502086

[31] Ma T, Zhang A (2019). Integrate multi-omics data with biological interaction networks using Multi-view Factorization AutoEncoder (MAE). BMC Genomics.

[32] Sharifi-Noghabi H, Zolotareva O, Collins CC, Ester M (2019). MOLI: multi-omics late integration with deep neural networks for drug response prediction. Bioinformatics.

[33] Tuncbag, N. et al. Network-based interpretation of diverse high-throughput datasets through the omics integrator software package. *PLoS Comput. Biol*. **12**, e1004879 (2016). 10.1371/journal.pcbi.1004879 PMC4838263 27096930

[34] Siddle K (2011). Signalling by insulin and IGF receptors: supporting acts and new players. J. Mol. Endocrinol..

[35] Saltiel AR, Kahn CR (2001). Insulin signalling and the regulation of glucose and lipid metabolism. Nature.

[36] Pollak M (2009). Insulin and insulin-like growth factor signalling in neoplasia. Nat. Rev. Cancer.

[37] Erdem, C. et al. Proteomic screening and Lasso regression reveal differential signaling in insulin and insulin-like growth factor I (IGF1) pathways. *Mol. Cell Proteom*. **15**, 3045–3057 (2016). 10.1074/mcp.M115.057729 PMC5013316 27364358

[38] Erdem C., Lee A. V., Taylor D. L., Lezon T. R. Inhibition of RPS6K reveals context-dependent Akt activity in luminal breast cancer cells. *PLoS Comput. Biol*. **17**:e1009125 (2021). 10.1371/journal.pcbi.1009125 PMC8277016 34191793

[39] Greene CS (2015). Understanding multicellular function and disease with human tissue-specific networks. Nat. Genet..

[40] Yang, H., Cao, H., He, T., Wang, T. & Cui, Y. Multilevel heterogeneous omics data integration with kernel fusion. *Brief Bioinformatics* **21**, 156–170 (2020). https://academic.oup.com/bib/advance-article/doi/10.1093/bib/bby115/5200557. 10.1093/bib/bby115 30496340

[41] Mariette, J. & Villa-Vialaneix, N. Unsupervised multiple kernel learning for heterogeneous data integration. *Bioinformatics* **15**, 1009–1015 (2018). 10.1093/bioinformatics/btx682 29077792

[42] Lin D (2013). Group sparse canonical correlation analysis for genomic data integration. BMC Bioinformatics.

[43] Jendoubi T, Strimmer K (2019). A whitening approach to probabilistic canonical correlation analysis for omics data integration. BMC Bioinformatics.

[44] Qi L (2021). Multi-omics data fusion for cancer molecular subtyping using sparse canonical correlation analysis. Front. Genet..

[45] Min, W., Chang, T. H., Zhang, S. & Wan, X. TSCCA: a tensor sparse CCA method for detecting microRNA-gene patterns from multiple cancers. *PLoS Comput. Biol*. **17**, e1009044 (2021). 10.1371/journal.pcbi.1009044 PMC8195367 34061840

[46] Hulot A, Laloë D, Jaffrézic F (2021). A unified framework for the integration of multiple hierarchical clusterings or networks from multi-source data. BMC Bioinformatics.

[47] Jansen R (2003). A Bayesian networks approach for predicting protein-protein interactions from genomic data. Science.

[48] Mo Q (2018). A fully Bayesian latent variable model for integrative clustering analysis of multi-type omics data. Biostatistics.

[49] Qian L, Zhou G (2012). Tree kernel-based protein–protein interaction extraction from biomedical literature. J. Biomed. Inf..

[50] Park, C., Ahn, J., Kim, H. & Park, S. Integrative gene network construction to analyze cancer recurrence using semi-supervised learning. *PLoS ONE* **9**, e86309 (2014). 10.1371/journal.pone.0086309 PMC3908883 24497942

[51] Lewis JE, Kemp ML (2021). Integration of machine learning and genome-scale metabolic modeling identifies multi-omics biomarkers for radiation resistance. Nat. Commun..

[52] Drake JM (2016). Phosphoproteome integration reveals patient-specific networks in prostate cancer. Cell..

[53] Paull EO (2013). Discovering causal pathways linking genomic events to transcriptional states using Tied Diffusion Through Interacting Events (TieDIE). Bioinformatics.

[54] Park CY (2015). Tissue-aware data integration approach for the inference of pathway interactions in metazoan organisms. Bioinformatics.

[55] Yao V (2018). An integrative tissue-network approach to identify and test human disease genes. Nat. Biotechnol..

[56] Basha, O. et al. Differential network analysis of multiple human tissue interactomes highlights tissue-selective processes and genetic disorder genes. *Bioinformatics* **36**, 2821–2828 (2020). 10.1093/bioinformatics/btaa034 31960892

[57] Ideker T, Krogan NJ (2012). Differential network biology. Mol. Syst. Biol..

[58] Xie J (2020). DNF: a differential network flow method to identify rewiring drivers for gene regulatory networks. Neurocomputing.

[59] Gill R, Datta S, Datta S (2010). A statistical framework for differential network analysis from microarray data. BMC Bioinformatics.

[60] Jardim VC, Santos S, de S, Fujita A, Buckeridge MS (2019). BioNetStat: a tool for biological networks differential analysis. Front. Genet..

[61] Goenawan IH, Bryan K, Lynn DJ (2016). DyNet: visualization and analysis of dynamic molecular interaction networks. Bioinformatics.

[62] Lichtblau, Y. et al Comparative assessment of differential network analysis methods. *Brief Bioinformatics* **18**, 837–850 (2016). 10.1093/bib/bbw061 27473063

[63] Bandyopadhyay S (2010). Rewiring of genetic networks in response to DNA damage. Science.

[64] Gill R, Datta S, Datta S (2014). Differential network analysis in human cancer research. Curr. Pharm. Des..

[65] Basha O, Shpringer R, Argov CM, Yeger-Lotem E (2018). The DifferentialNet database of differential protein–protein interactions in human tissues. Nucleic Acids Res..

[66] Grimes T, Potter SS, Datta S (2019). Integrating gene regulatory pathways into differential network analysis of gene expression data. Sci. Rep..

[67] Ji, J. et al. JDINAC: joint density-based non-parametric differential interaction network analysis and classification using high-dimensional sparse omics data. *Bioinformatics* **33**,3080–3087 (2017). 10.1093/bioinformatics/btx360 PMC5870609 28582486

[68] Ruan D, Young A, Montana G (2015). Differential analysis of biological networks. BMC Bioinformatics.

[69] Mall, R. et al. Differential community detection in paired biological networks. In *Proc. 8th ACM International Conference on Bioinformatics, Computational Biology, and Health Informatics* 330–339 (ACM; 2017).

[70] Levi, H., Elkon, R. & Shamir, R. DOMINO: a network‐based active module identification algorithm with reduced rate of false calls. *Mol. Syst. Biol.* **17**, e9593 (2021). 10.15252/msb.20209593 PMC7816759 33471440

[71] Argelaguet, R. et al. Multi‐omics factor analysis—a framework for unsupervised integration of multi‐omics data sets. *Mol. Syst. Biol.* **17**, e95932018 (2021). https://onlinelibrary.wiley.com/doi/10.15252/msb.20178124. 10.15252/msb.20178124 PMC6010767 29925568

[72] Gomez-Cabrero D (2014). Data integration in the era of omics: current and future challenges. BMC Syst. Biol..

[73] Huang S, Hu P, Lakowski TM (2021). Predicting breast cancer drug response using a multiple-layer cell line drug response network model. BMC Cancer.

[74] Yu MK (2018). Visible machine learning for biomedicine. Cell.

[75] AlQuraishi M, Sorger PK (2021). Differentiable biology: using deep learning for biophysics-based and data-driven modeling of molecular mechanisms. Nat. Methods.

[76] Gross SM (2022). A multi-omic analysis of MCF10A cells provides a resource for integrative assessment of ligand-mediated molecular and phenotypic responses. Commun. Biol..

[77] Lin JR, Fallahi-Sichani M, Sorger PK (2015). Highly multiplexed imaging of single cells using a high-throughput cyclic immunofluorescence method. Nat. Commun..

[78] Tibes R (2006). Reverse phase protein array: validation of a novel proteomic technology and utility for analysis of primary leukemia specimens and hematopoietic stem cells. Mol. Cancer Ther..

[79] Tibshirani R (1996). Regression shrinkage and selection via the Lasso. J. R. Stat. Soc. Ser. B Methodol..

[80] Subramanian A (2005). Gene set enrichment analysis: a knowledge-based approach for interpreting genome-wide expression profiles. Proc. Natl Acad. Sci. USA.

[81] Jassal B (2020). The reactome pathway knowledgebase. Nucleic Acids Res.

[82] Wells CM, Ahmed T, Masters JRW, Jones GE (2005). Rho family GTPases are activated during HGF-stimulated prostate cancer-cell scattering. Cell Motil. Cytoskeleton.

[83] Ye IC (2018). Molecular portrait of hypoxia in breast cancer: a prognostic signature and novel HIF-regulated genes. Mol. Cancer Res..

[84] Subramanian DN (2020). Exome sequencing of familial high-grade serous ovarian carcinoma reveals heterogeneity for rare candidate susceptibility genes. Nat. Commun..

[85] Kinyamu HK, Collins JB, Grissom SF, Hebbar PB, Archer TK (2008). Genome wide transcriptional profiling in breast cancer cells reveals distinct changes in hormone receptor target genes and chromatin modifying enzymes after proteasome inhibition. Mol. Carcinog..

[86] Porter JD (2006). Distinctive morphological and gene/protein expression signatures during myogenesis in novel cell lines from extraocular and hindlimb muscle. Physiol. Genomics.

[87] Wang S (2016). Modeling *cis* -regulation with a compendium of genome-wide histone H3K27ac profiles. Genome Res..

[88] Arya KR (2021). Identification of Hub genes and key pathways associated with anti-VEGF resistant glioblastoma using gene expression data analysis. Biomolecules.

[89] Mourksi NEH, Morin C, Fenouil T, Diaz JJ, Marcel V (2020). snoRNAs offer novel insight and promising perspectives for lung cancer understanding and management. Cells.

[90] Kinchen J (2018). Structural remodeling of the human colonic mesenchyme in inflammatory bowel disease. Cell..

[91] Fang Y (2003). PLD1 regulates mTOR signaling and mediates Cdc42 activation of S6K1. Curr. Biol..

[92] Endo M, Antonyak MA, Cerione RA (2009). Cdc42-mTOR signaling pathway controls Hes5 and Pax6 expression in retinoic acid-dependent neural differentiation. J. Biol. Chem..

[93] Ye, H. et al. The m6A writers regulated by the IL-6/STAT3 inflammatory pathway facilitate cancer cell stemness in cholangiocarcinoma. *Cancer Biol. Med*. **19**, 343–357 (2021). 10.20892/j.issn.2095-3941.2020.0661 PMC8958887 34347395

[94] Yu Y, Feng XH (2019). TGF-β signaling in cell fate control and cancer. Curr. Opin. Cell Biol..

[95] Greten FR, Grivennikov SI (2019). Inflammation and cancer: triggers, mechanisms, and consequences. Immunity.

[96] Zhao H (2021). Inflammation and tumor progression: signaling pathways and targeted intervention. Signal Transduct. Target Ther..

[97] Ju X, Zhang H, Zhou Z, Wang Q (2020). Regulation of PD-L1 expression in cancer and clinical implications in immunotherapy. Am. J. Cancer Res..

[98] Gong J, Chehrazi-Raffle A, Reddi S, Salgia R (2018). Development of PD-1 and PD-L1 inhibitors as a form of cancer immunotherapy: a comprehensive review of registration trials and future considerations. J. Immunother. Cancer.

[99] Wu Y, Chen W, Xu ZP, Gu W (2019). PD-L1 distribution and perspective for cancer immunotherapy—Blockade, knockdown, or inhibition. Front. Immunol..

[100] Thiem A (2019). IFN-gamma-induced PD-L1 expression in melanoma depends on p53 expression. J. Exp. Clin. Cancer Res..

[101] Abril-Rodriguez G, Ribas A (2017). SnapShot: immune checkpoint inhibitors. Cancer Cell.

[102] Alsaab HO (2017). PD-1 and PD-L1 checkpoint signaling inhibition for cancer immunotherapy: mechanism, combinations, and clinical outcome. Front. Pharm..

[103] Wang, Y. et al. Anti-PD-1/L1 lead-in before MAPK inhibitor combination maximizes antitumor immunity and efficacy. *Cancer Cell* **39**, 1375–87.e6 (2021). 10.1016/j.ccell.2021.07.023 PMC9126729 34416167

[104] Mei Y, Chen MJM, Liang H, Ma L (2021). A four-gene signature predicts survival and anti-CTLA4 immunotherapeutic responses based on immune classification of melanoma. Commun. Biol..

[105] Xu T (2020). Chloride intracellular channel protein 2: prognostic marker and correlation with PD-1/PD-L1 in breast cancer. Aging.

[106] Wang Z (2013). FAM83D promotes cell proliferation and motility by downregulating tumor suppressor gene FBXW7. Oncotarget.

[107] Walian PJ, Hang B, Mao JH (2016). Prognostic significance of FAM83D gene expression across human cancer types. Oncotarget.

[108] Huang M (2017). *FAM83D*, a microtubule-associated protein, promotes tumor growth and progression of human gastric cancer. Oncotarget.

[109] Zhou F, Wang X, Liu F, Meng Q, Yu Y (2020). FAM83A drives PD-L1 expression via ERK signaling and FAM83A/PD-L1 co-expression correlates with poor prognosis in lung adenocarcinoma. Int. J. Clin. Oncol..

[110] Akhurst RJ, Hata A (2012). Targeting the TGFβ signalling pathway in disease. Nat. Rev. Drug Discov..

[111] Massagué J (2012). TGFβ signalling in context. Nat. Rev. Mol. Cell Biol..

[112] Nickel J, Mueller TD (2019). Specification of BMP signaling. Cells.

[113] Derynck R, Budi EH (2019). Specificity, versatility, and control of TGF-β family signaling. Sci. Signal.

[114] Caestecker MPde (2000). Role of transforming growth factor-beta signaling in cancer. J. Natl Cancer Inst..

[115] Derynck R, Turley SJ, Akhurst RJ (2021). TGFβ biology in cancer progression and immunotherapy. Nat. Rev. Clin. Oncol..

[116] Guo X, Wang XF (2009). Signaling cross-talk between TGF-β/BMP and other pathways. Cell Res..

[117] Rahman MS, Akhtar N, Jamil HM, Banik RS, Asaduzzaman SM (2015). TGF-β/BMP signaling and other molecular events: regulation of osteoblastogenesis and bone formation. Bone Res..

[118] Zhang YE (2009). Non-Smad pathways in TGF-β signaling. Cell Res.

[119] Wu L, Derynck R (2009). Essential role of TGF-beta signaling in glucose-induced cell hypertrophy. Dev. Cell.

[120] Lamouille S, Derynck R (2007). Cell size and invasion in TGF-β–induced epithelial to mesenchymal transition is regulated by activation of the mTOR pathway. J. Cell Biol..

[121] Mootha VK (2003). PGC-1α-responsive genes involved in oxidative phosphorylation are coordinately downregulated in human diabetes. Nat. Genet.

[122] Miyazaki K (2006). Laminin-5 (laminin-332): unique biological activity and role in tumor growth and invasion. Cancer Sci..

[123] Miller KA (2000). Inhibition of laminin-5 production in breast epithelial cells by overexpression of p300. J. Biol. Chem..

[124] Aberdam D, Virolle T, Simon-Assmann P (2000). Transcriptional regulation of laminin gene expression. Microsc. Res. Tech..

[125] Korang K, Christiano AM, Uitto J, Mauviel A (1995). Differential cytokine modulation of the genes LAMA3, LAMB3, and LAMC2, encoding the constitutive polypeptides, alpha 3, beta 3, and gamma 2, of human laminin 5 in epidermal keratinocytes. FEBS Lett..

[126] Virolle T (1998). Three activator protein-1-binding sites bound by the Fra-2·JunD complex cooperate for the regulation of murine laminin α3A (lama3A) promoter activity by transforming growth factor-β. J. Biol. Chem..

[127] Domogatskaya A, Rodin S, Tryggvason K (2012). Functional diversity of laminins. Annu. Rev. Cell Dev. Biol..

[128] Rousselle P (1997). Laminin 5 binds the NC-1 domain of type VII collagen. J. Cell Biol..

[129] Gonzales M (1999). A cell signal pathway involving laminin-5, alpha3beta1 integrin, and mitogen-activated protein kinase can regulate epithelial cell proliferation. Mol. Biol. Cell.

[130] Ryan MC, Lee K, Miyashita Y, Carter WG (1999). Targeted disruption of the LAMA3 gene in mice reveals abnormalities in survival and late stage differentiation of epithelial cells. J. Cell Biol..

[131] Vazquez-Martin A, Ortega-Delgado FJ, Fernandez-Real JM, Menendez JA (2008). The tyrosine kinase receptor HER2 (erbB-2): from oncogenesis to adipogenesis. J. Cell Biochem..

[132] Antalis CJ (2010). High ACAT1 expression in estrogen receptor negative basal-like breast cancer cells is associated with LDL-induced proliferation. Breast Cancer Res. Treat..

[133] Kim S, Lee Y, Koo JS (2015). Differential expression of lipid metabolism-related proteins in different breast cancer subtypes. PLoS ONE.

[134] Wang L, Zhang S, Wang X (2021). The metabolic mechanisms of breast cancer metastasis. Front. Oncol..

[135] Ligorio F (2021). Targeting lipid metabolism is an emerging strategy to enhance the efficacy of anti-HER2 therapies in HER2-positive breast cancer. Cancer Lett..

[136] Santamaria S (2021). Imaging of endocytic trafficking and extracellular vesicles released under neratinib treatment in ERBB2+ breast cancer cells. J. Histochem. Cytochem..

[137] Su JC (2016). Disrupting VEGF-A paracrine and autocrine loops by targeting SHP-1 suppresses triple negative breast cancer metastasis. Sci. Rep..

[138] Wang C (2018). Oestrogen inhibits VEGF expression and angiogenesis in triple-negative breast cancer by activating GPER-1. J. Cancer.

[139] Pohl SG (2017). Wnt signaling in triple-negative breast cancer. Oncogenesis.

[140] Merikhian P, Eisavand MR, Farahmand L (2021). Triple-negative breast cancer: understanding Wnt signaling in drug resistance. Cancer Cell Int..

[141] Shen N (2021). DLST-dependence dictates metabolic heterogeneity in TCA-cycle usage among triple-negative breast cancer.. Commun. Biol..

[142] Sun, X. et al. Metabolic reprogramming in triple-negative breast cancer. *Front. Oncol.* **10**, 428 (2020). 10.3389/fonc.2020.00428 PMC7136496 32296646

[143] Delgir S (2021). The pathways related to glutamine metabolism, glutamine inhibitors and their implication for improving the efficiency of chemotherapy in triple-negative breast cancer. Mutat Res. Mutat. Res..

[144] Miller TW, Rexer BN, Garrett JT, Arteaga CL (2011). Mutations in the phosphatidylinositol 3-kinase pathway: role in tumor progression and therapeutic implications in breast cancer. Breast Cancer Res..

[145] Fu X, Osborne CK, Schiff R (2013). Biology and therapeutic potential of PI3K signaling in ER+/HER2-negative breast cancer. Breast.

[146] Bosch A (2015). PI3K inhibition results in enhanced estrogen receptor function and dependence in hormone receptor-positive breast cancer. Sci. Transl. Med..

[147] du Rusquec P, Blonz C, Frenel JS, Campone M (2020). Targeting the PI3K/Akt/mTOR pathway in estrogen-receptor positive HER2 negative advanced breast cancer. Ther. Adv. Med. Oncol..

[148] Shao C, Folkard M, Held KD, Prise KM (2008). Estrogen enhanced cell-cell signalling in breast cancer cells exposed to targeted irradiation. BMC Cancer.

[149] Ungerleider NA (2018). Breast cancer survival predicted by TP53 mutation status differs markedly depending on treatment. Breast Cancer Res.

[150] Das K (2009). Positive association between nuclear Runx2 and oestrogen-progesterone receptor gene expression characterises a biological subtype of breast cancer. Eur. J. Cancer.

[151] Chang CH (2014). The prognostic significance of RUNX2 and miR-10a/10b and their inter-relationship in breast cancer. J. Transl. Med.

[152] Hsu LH, Chu NM, Lin YF, Kao SH (2019). G-protein coupled estrogen receptor in breast cancer. Int. J. Mol. Sci..

[153] Luo, J. & Liu, D. Does GPER really function as a G protein-coupled estrogen receptor in vivo? *Front. Endocrinol.* **11**, 148 (2020). https://www.frontiersin.org/articles/10.3389/fendo.2020.00148. 10.3389/fendo.2020.00148 PMC7137379 32296387

[154] Prossnitz ER (2008). Estrogen signaling through the transmembrane G protein–coupled receptor GPR30. Annu. Rev. Physiol..

[155] Bratton MR (2012). Gαo potentiates estrogen receptor α activity via the ERK signaling pathway. J. Endocrinol..

[156] Lappano R, Jacquot Y, Maggiolini M (2018). GPCR modulation in breast cancer. Int. J. Mol. Sci..

[157] Barretina J (2012). The Cancer Cell Line Encyclopedia enables predictive modelling of anticancer drug sensitivity. Nature.

[158] Rohart, F., Gautier, B., Singh, A., & Lê, Cao K. A. mixOmics: an R package for ‘omics feature selection and multiple data integration. *PLoS Comput. Biol*. **13**, e1005752 (2017). 10.1371/journal.pcbi.1005752 PMC5687754 29099853

[159] McArdle, S. et al. PRESTO, a new tool for integrating large-scale -omics data and discovering disease-specific signatures. *Bioinformatics* **35**, i191–i199 (2019).

[160] Meng C (2019). MOGSA: integrative single sample gene-set analysis of multiple omics data. Mol. Cell Proteom..

[161] Qian Hastie, T., Friedman, J., Tibshirani, R. & Simon, N. J. Glmnet for Matlab. http://www.stanford.edu/hastie/glmnet_matlab/ (2013).

[162] Shannon P (2003). Cytoscape: a software environment for integrated models of biomolecular interaction networks. Genome Res..

[163] Doncheva NT, Morris JH, Gorodkin J, Jensen LJ (2019). Cytoscape StringApp: network analysis and visualization of proteomics data. J. Proteome Res..

[164] Erdem C (2022). A scalable, open-source implementation of a large-scale mechanistic model for single cell proliferation and death signaling. Nat. Commun..

[165] BirtwistleLab, Erdem C. SourceData_MOBILE. figshare https://figshare.com/articles/dataset/Source_Data_-_MOBILE/20294229 (2023).

[166] Erdem, Cemal. MOBILE. *Zenodo.* https://zenodo.org/record/7764731 (2023).

