# Supplementary material for: MOBILE pipeline enables identification of context-specific networks and regulatory mechanisms
Source: Nat Commun. 2023 Jul 6;14:3991. doi: 10.1038/s41467-023-39729-2 (PMC10326020; doi:10.1038/s41467-023-39729-2)
Supplement: Supplementary file 1 — Supplementary Information [file 41467_2023_39729_MOESM1_ESM.pdf]

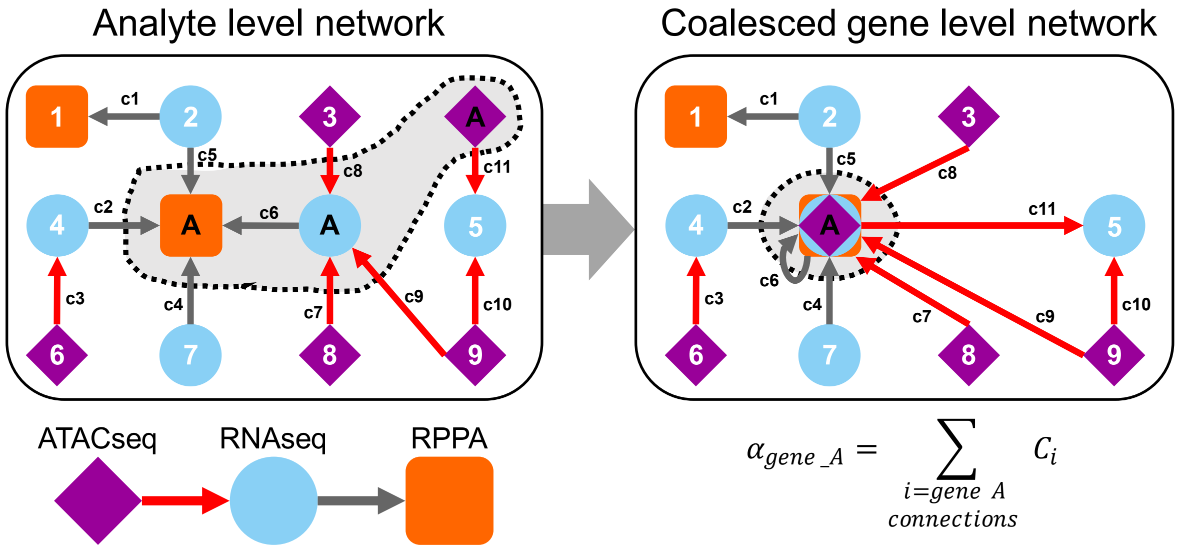

Supplementary Fig. 1 The analyte level association matrices are used to create gene-level coalescence networks. Different analytes (ATACseq: diamond, RNAseq: circle, RPPA: square) can have the same gene. For instance, gene A has protein, RNA, and ATACseq peak nodes, each associated with one or more other nodes. The numbers (1-9) represent different nodes corresponding to different genes besides gene A. The analytes with the same gene (i.e., A) are coalesced into a single node, preserving all the edges. After coalescence of all such nodes, the weights (MOBILE inferred association coefficients) of all incoming edges are summed to create a ranked gene-list of the network. Red arrows represent association edges between ATACseq-RNAseq analytes, and the black arrows represent association edges between RNAseq-RPPA nodes. The RPPA analytes can represent phospho- or total protein level measurements.

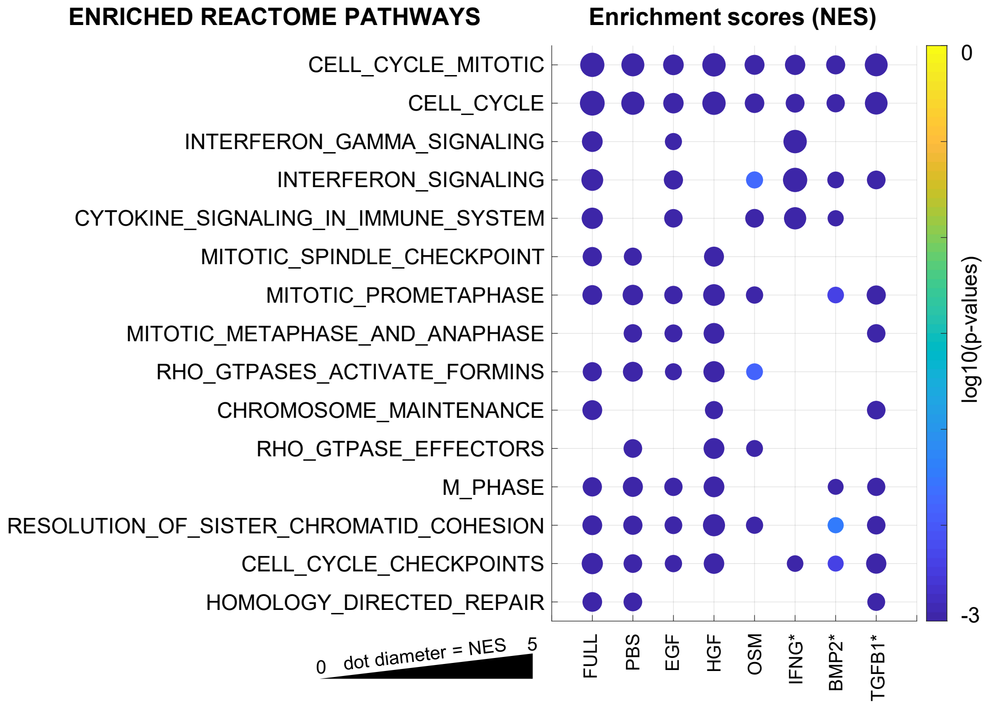

Supplementary Fig. 2 MOBILE inferred IANs are enriched for canonical pathways and the top associations are literature verified interactions. Gene set enrichment analysis using genes of FULL and ligand dependent associations reveal cell cycle, interferon, and cytokine signaling pathways. Only top 15 significantly enriched pathways (ranked based on mean scores across conditions) are shown (p-values<0.05, FDR<0.1, significance tested using default GSEA settings of single-tail null distributions (80)). The dot diameter corresponds to the normalized enrichment scores (NES) and color denotes p-values (one-sided test with multiple comparison adjustment). Asterisk in column labels denotes conditions with additional EGF treatment.

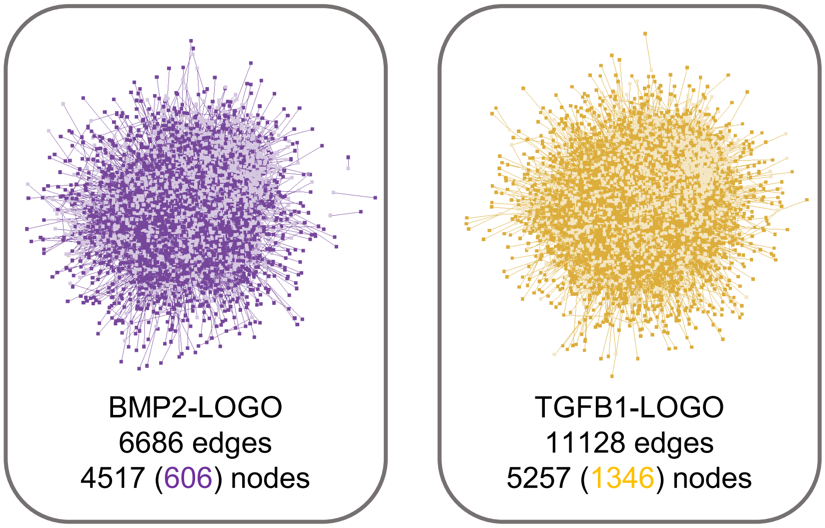

Supplementary Fig. 3 Comparison of TGFβ1- and BMP2-IANs to FULL network reveals distinct nodes & edges. There are thousands of ligand-specific associations (number of edges) when the Integrated Association Networks (IANs) of the TGFβ1 and BMP2 are compared. The genes within these sub-networks are ranked by weighted sum of edge magnitudes. Colored numbers represent the number of ligand-specific genes (nodes) in the corresponding networks.

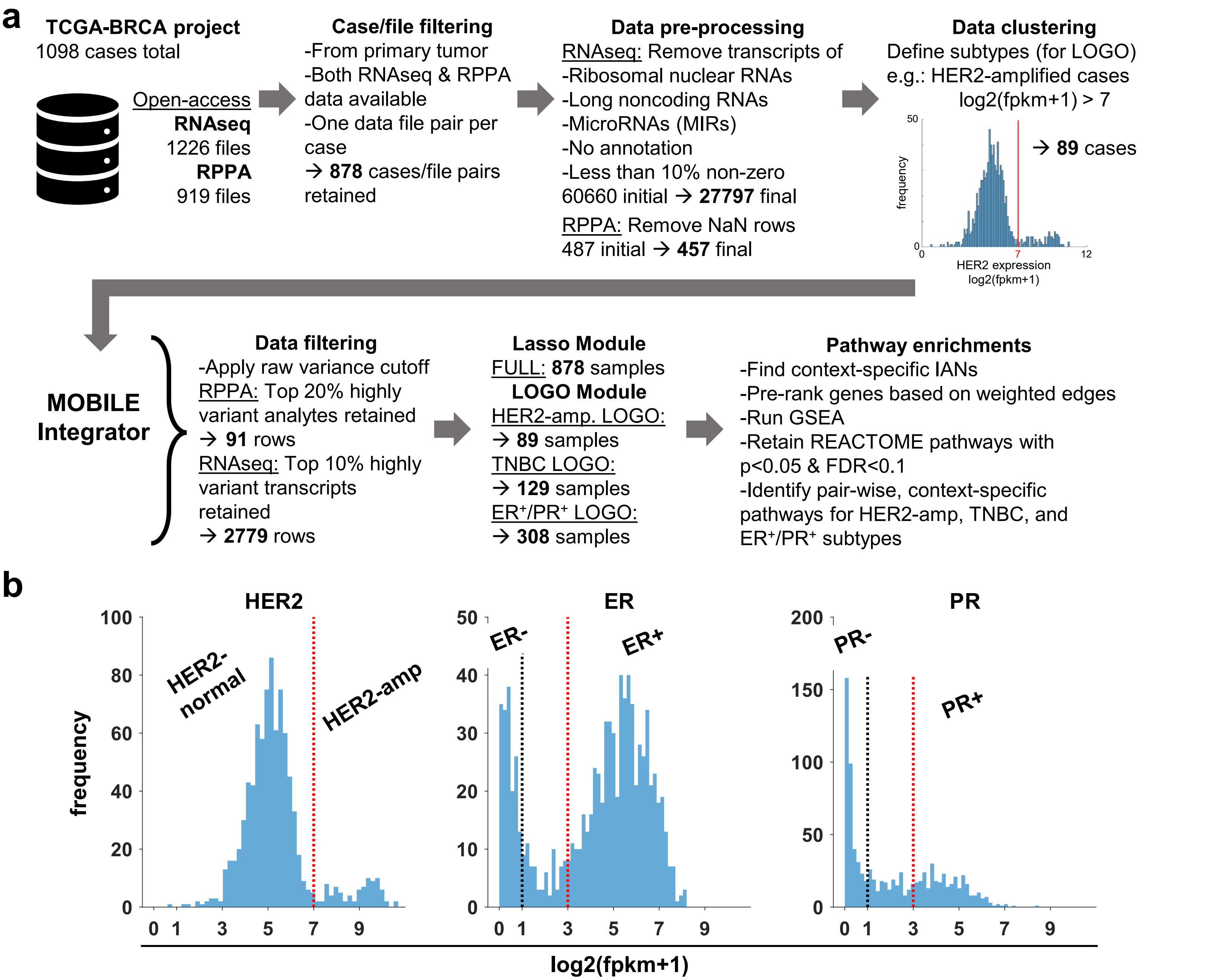

Supplementary Fig. 4 Filtering TCGA data and empirical determination of breast cancer (BRCA) subtypes based on transcriptomic data. a Transcriptomic and proteomic datasets of TCGA breast cancer (BRCA) project are downloaded and filtered to obtain paired data files per case/sample. 878/1098 cases are represented in the final list. The RNAseq and RPPA data are further cleaned and finalized to contain 27797 transcript levels (RNAseq) and 457 protein levels (RPPA). Based on transcript levels, HER2-amplified, triple-negative (TNBC), and estrogen and progesterone receptor positive (ER+/PR+) subtypes are determined. Then, as per MOBILE pipeline, only the top highly variant analytes are retained for both assay types and the MOBILE Lasso Module is run using all samples (878 columns) to obtain the FULL IAN. Next, the sample columns for each subtype are excluded from the input data and LOGO module is run to obtain context-specific IANs. **b** HER2-amplified (HER2amp), triple-negative (ER-, PR-, and HER2-normal), and ER+/PR+ are the three subtypes analyzed. The red dashed lines represent the cut-off for receptor-amplified and receptor-positive samples and black dashed lines show the cut-off for receptor-normal and receptor-negative samples.

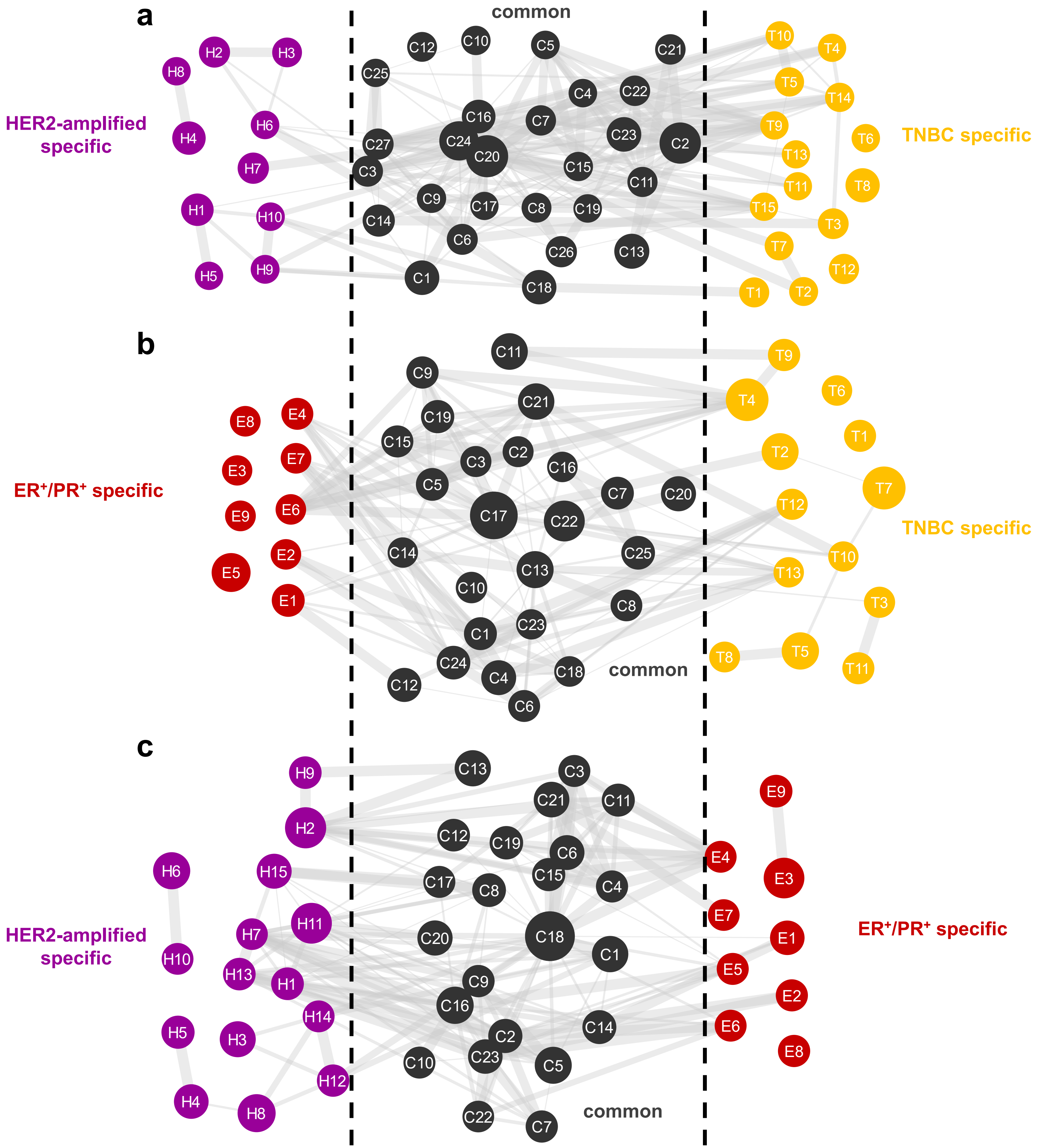

Supplementary Fig. 5 MOBILE infers breast cancer subtype specific pathways based on pair-wise comparisons. a HER2-amplified vs TNBC differentially enriched pathways. **b** ER^+/PR^+ vs TNBC differentially enriched pathways. **c** HER2-amplified vs ER^+/PR^+ differentially enriched pathways. Node size and color represent gene-set size and subtype, respectively. The edge widths represent gene-set similarity coefficients of the connected nodes. The pathways outside the dashed lines are the ones used to visualize Fig. 7 network. The names of Reactome pathways are given in Supplementary Data 16-19.

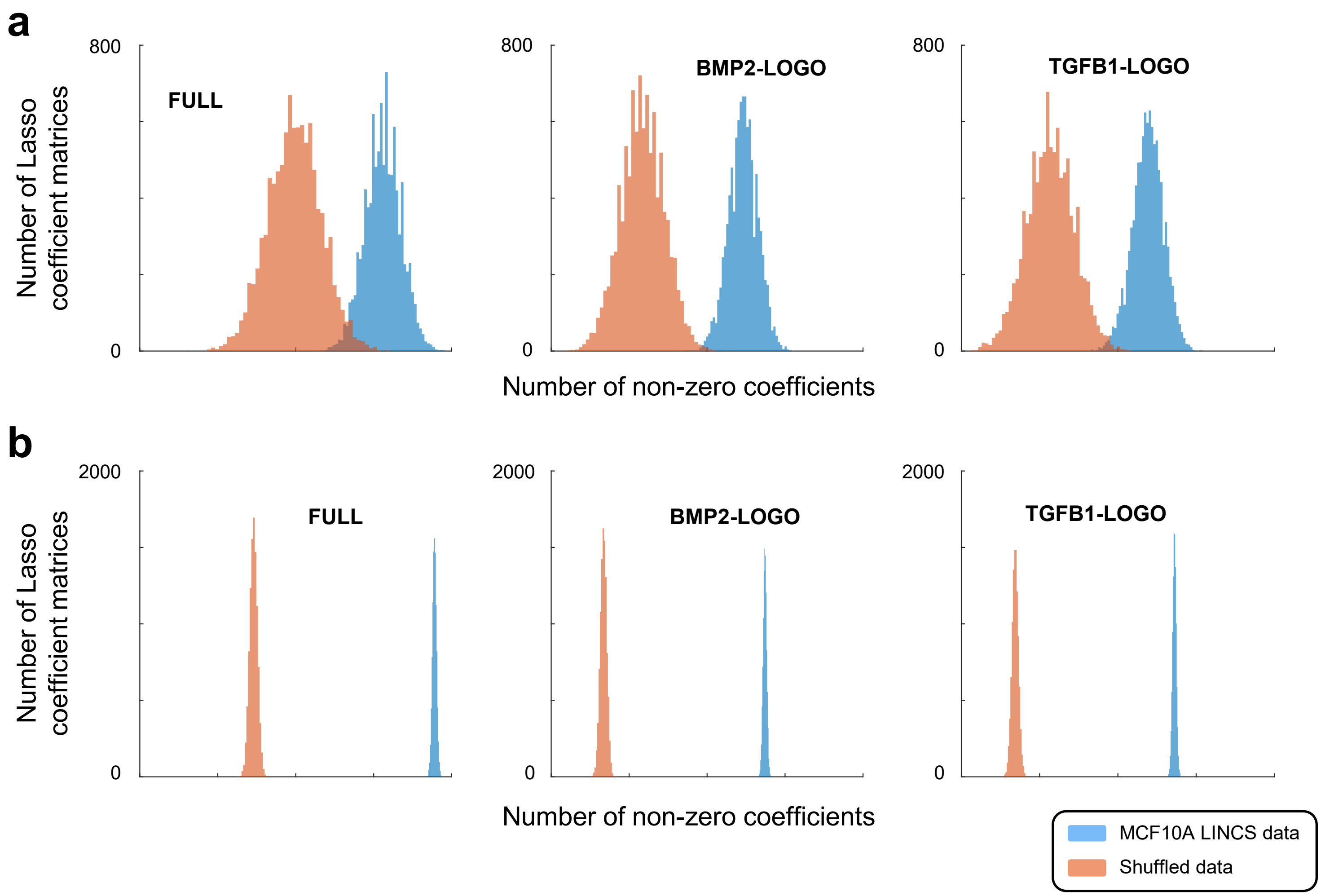

Supplementary Fig. 6 MOBILE infers significantly different number of Lasso coefficients in real (LINCS) data compared to shuffled (randomized) input. a Histograms of the Lasso coefficient matrices and their non-zero coefficients from RNAseq-RPPA pair of inputs. Real data input yields more non-zero coefficients inferred, partly due to larger information content. Also note that the FULL data input histograms (top left) are shifted to the right compared to the LOGO conditions. **b** Histograms from the ATACseq-RNAseq pair of input matrices.

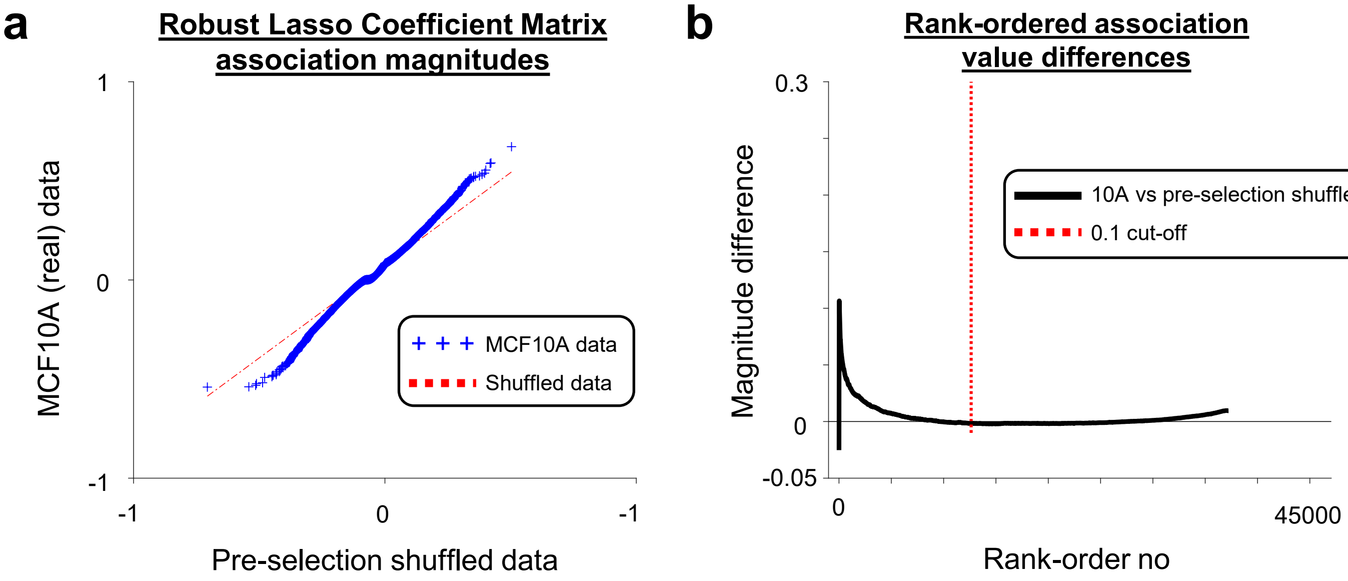

Supplementary Fig. 7 The magnitude cutoff determination based on real and shuffled datasets. a The QQ-plots of real vs. shuffled data show skewed trends at the tails of real data associations. The divergence from shuffled data quantiles starts around 0.1 and -0.1. **b** The magnitude difference between real and shuffled data coefficients in ordered lists of associations smoothens around an absolute value of 0.1.

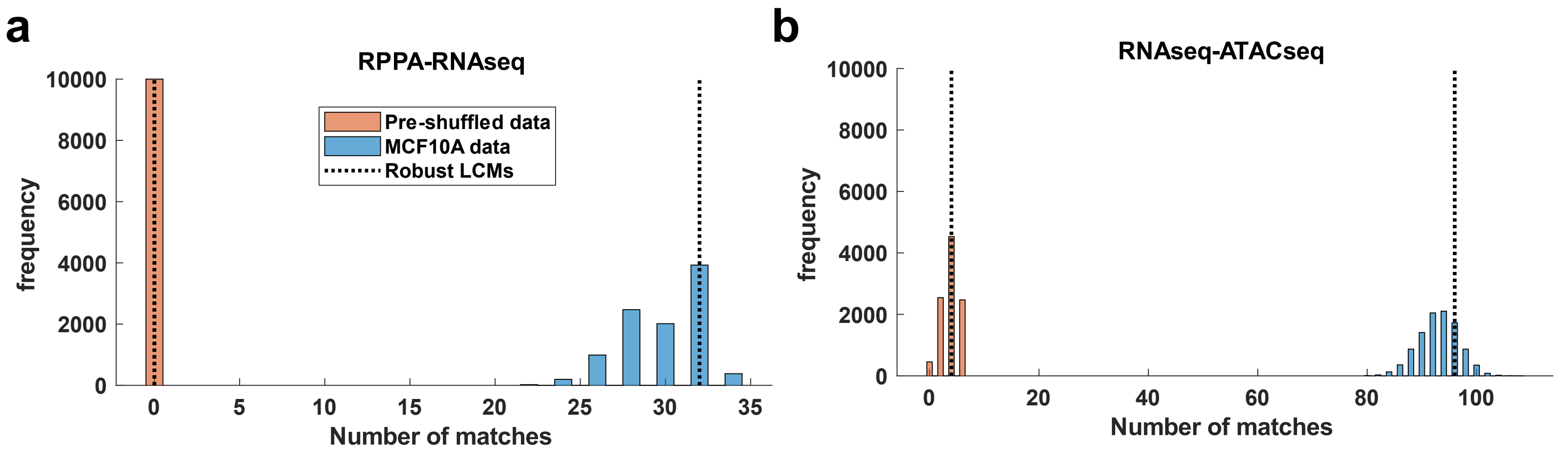

Supplementary Fig. 8 MOBILE inferred associations vs literature-based interactions in real and shuffled data cases. The genes from the RPPA, RNAseq, and ATACseq input matrices are used to obtain literature-based associations in stringApp (164) within Cytoscape (163) (Supplementary Data 20-21). Then, the overlap between 10000 Lasso coefficient matrices of real (blue distributions) and shuffled (orange distributions) data are determined. **a** In the RPPA-RNAseq input case, the shuffled datasets could not infer known interactions. The real data input however showed over 20 interactions in each Lasso matrix and the final Robust Lasso Coefficient Matrix (Robust LCM). **b** In RNAseq-ATACseq input case, the shuffled Lasso matrices showed a low number of known interaction predictions. Compared to shuffled data, the overall signal-to-noise ratio is around 19:1. The dashed lines show the number of inferred literature associations in the Robust LCMs.
