# Supplementary material for: MOBILE pipeline enables identification of context-specific networks and regulatory mechanisms
Source: Nat Commun. 2023 Jul 6;14:3991. doi: 10.1038/s41467-023-39729-2 (PMC10326020; doi:10.1038/s41467-023-39729-2)
Supplement: Supplementary file 3 — Editorial Assessment Report [file 41467_2023_39729_MOESM3_ESM.pdf]

nature portfolio GUIDED OPEN ACCESS

Contents of this report

1. **Manuscript details**: overview of your manuscript and the editorial team.
2. **Review synthesis**: summary of the reviewer reports provided by the editors.
3. **Editorial recommendation**: personalized evaluation and recommendation from all 3 journals.
4. **Annotated reviewer comments**: the referee reports with comments from the editors.
5. **Open research evaluation**: advice for adhering to best reproducibility practices.

About the editorial process

Because you selected the **Nature Portfolio Guided Open Access** option, your manuscript was assessed for suitability in three of our titles publishing high-quality work in your field of research. More information about Guided Open Access can be found here.

Collaborative editorial assessment

Your editorial team discussed the manuscript to determine its suitability for the Nature Portfolio Guided OA pilot. Our assessment of your manuscript takes into account several factors, including whether the work meets the technical standard of the Nature Portfolio and whether the findings are of immediate significance to the readership of at least one of the participating journals in the Guided OA pilot.

Peer review

Experts were asked to evaluate the following aspects of your manuscript:

- **Novelty** in comparison to prior publications;
- **Likely audience** of researchers in terms of broad fields of study and size;
- **Potential impact** of the study on the immediate or wider research field;
- **Evidence** for the claims and whether additional experiments or analyses could feasibly strengthen the evidence;
- **Methodological detail** and whether the manuscript is reproducible as written;
- Appropriateness of the **literature review**.

Editorial evaluation of reviews

Your editorial team discussed the potential suitability of your manuscript for each of the participating journals. They then discussed the revisions necessary in order for the work to be published, keeping each journal's specific editorial criteria in mind.

Journals in the Nature portfolio will support authors wishing to transfer their reviews and (where reviewers agree) the reviewers' identities to journals outside of Springer Nature.
If you have any questions about review portability, please contact our editorial office at guidedoa@nature.com.

nature portfolio GUIDED OPEN ACCESS

Manuscript details

Tracking number	Submission date	Decision date	Peer review type
GUIDEDOA-22-00469	**Jul 27, 2022**	**Sep 21, 2022**	Single-blind

Manuscript title

Multi-Omics Binary Integration via Lasso Ensembles (MOBILE) for identification of context-specific networks and new regulatory mechanisms

Preprint: BioRxiv

Author details

Marc Birtwistle

Affiliation: Clemson University

Editorial assessment team

**Primary editor**	**Cara Eldridge**<br>**Home journal:** *Nature Communications*<br>**ORCID:** 0000-0001-7001-2312<br>**Email:** cara.eldridge@nature.com
**Other editors consulted**	**Lin Tang**<br>**Home journal:** *Nature Methods*<br>**ORCID:** 0000-0002-6050-0424<br><br>**George Inglis**<br>**Home journal:** *Communications Biology*<br>**ORCID:** 0000-0002-9069-5242
**About your primary editor**	Cara joined *Nature Communications* in March 2020. After conducting her undergraduate and Master's degrees in Natural Sciences at the University of Cambridge, she went on to complete a PhD at the MRC-Laboratory of Molecular Biology and continued there as a postdoctoral researcher. The focus of her research has been on the effect of replication impediments on stem cell differentiation, and as part of this work she established human embryonic stem cells and induced pluripotent stem cells into the lab. She handles manuscripts in the areas of cellular biotechnology and methods, as well as imaging. Cara is based in the London office.

Editorial assessment and review synthesis

**Editor's summary and assessment**	Here the authors report a multi-omics data integration strategy called MOBILE (Multi-Omics Binary Integration via Lasso Ensembles) to nominate molecular features associated with specific cellular phenotypes. They use the recent multi-omics dataset generated by the NIH LINCS Consortium. They pair ATACseq-RNAseq and RNAseq-RPPA matrices and apply Lasso. They perform further validation to show that their method can recover known biology and extra meaningful ligand-specific associations. They then fit their findings into the literature for further validation. We found this to be a nice method which had the potential to generate new findings.
**Editorial synthesis of reviewer reports**	The reviewers agreed that the method was interesting, and had the potential to yield important and potentially novel insight.<br><br>Reviewer #2 has concerns about the advance over existing work, and felt a more thorough comparison to the literature would be appropriate. Reviewer #1 had concerns about the statistical analysis, which would need to be addressed in a revision. Both reviewers felt that this would be substantially improved with the addition of at least one other dataset, which the editorial team agrees with.

Editorial recommendation

Nature Methods<br><br>Revision not invited	Neither the conceptual advance nor advance in performance demonstrated is sufficient for publication in *Nature Methods*.
Nature Communications<br><br>Major revisions	*Nature Communications* would be interested in seeing a revised manuscript addressing the reviewer concerns (detailed below in the annotated reviewer report section), and adding in analysis of at least one additional dataset - ideally using patient samples as suggested by the reviewer.
Communications Biology<br><br>Minor revisions	*Communications Biology* would be similarly interested in a revised manuscript that addresses Reviewer #1's concern regarding potential false positives, and Reviewer #2's suggestions for an expanded overview of relevant methods or tools. While we would strongly encourage you to include additional case studies demonstrating the utility of this method, at an absolute minimum the current focus on MCF10A cells should be stated as a limitation in the main text.

Next steps

**Editorial recommendation 1:**	Our top recommendation is to revise and resubmit your manuscript to *Nature Communications.* We feel the additional experiments required are reasonable to perform within an extended timeframe.
**Editorial recommendation 2:**	You may also choose to revise and resubmit your manuscript to *Communications Biology.* This option might be best if the requested experimental revisions are not possible/feasible at this time and you would prefer to get the manuscript published quickly.
**Note**	As stated on the previous page *Nature Methods* is not inviting a revision at this time. Please keep in mind that the journal will not be able to consider any appeals of their decision through Guided Open Access.

Revision

To follow our recommendation, please upload the revised manuscript files using **the link provided in the decision letter**. Should you need assistance with our manuscript tracking system, please contact Adam Lipkin, our Nature Portfolio Guided OA support specialist, at guidedOA@nature.com.

Revision checklist

- ☐ Cover letter, stating to which journal you are submitting
- ☐ Revised manuscript
- ☐ Point-by-point response to reviews
- ☐ Updated Reporting Summary and Editorial Policy Checklist
- ☐ Supplementary materials (if applicable)

Submission elsewhere

If you choose not to follow our recommendations, you can still take the reviewer reports with you.

Option 1: Transfer to another Nature Portfolio journal
Springer Nature provides authors with the ability to transfer a manuscript within the Nature Portfolio, without the author having to upload the manuscript data again. To use this service, **please follow the transfer link provided in the decision letter.** If no link was provided, please contact guidedOA@nature.com.

> *Note that any decision to opt in to In Review at the original journal is not sent to the receiving journal on transfer. You can opt in to In Review at receiving journals that support this service by choosing to modify your manuscript on transfer.*

Option 2: Portable Peer Review option for submission to a journal outside of Nature Portfolio
If you choose to submit your revised manuscript to a journal at another publisher, we can share the reviews with another journal outside of the Nature Portfolio if requested. You will need to request that the receiving journal office contacts us at guidedOA@nature.com. We have included editorial guidance below in the reviewer reports and open research evaluation to aid in revising the manuscript for publication elsewhere.

Annotated reviewer reports

The editors have included some additional comments on specific points raised by the reviewers below, to clarify requirements for publication in the recommended journal(s). However, please note that all points should be addressed in a revision, even if an editor has not specifically commented on them.

Reviewer #1 information	
**Expertise**	Interaction networks; pathway analysis; multi-omics; discovery of new biomarkers; machine learning
**Editor's comments**	This reviewer finds your manuscript to report interesting results, and finds the method to be broadly useful. Their main concerns are with the use of statistics, and data interpretation; they also find that your manuscript would benefit from additional case studies using a wider range of datasets.

Reviewer #1 comments	
**Section**	**Annotated Reviewer Comments**
**Remarks to the Author**	In this manuscript, the authors developed a multi-omics data integration method that uses ensemble lasso regression on pairs of biologically informed datasets. The authors first integrated ATACseq-RNAseq and RNAseq-RPPA matrices from a MCF10 LINCS Consortium dataset that reflects a series of ligand perturbations of the MCF10A cell line. Using this analysis, they produced a ligand-specific gene association network and examined one slice of this data representing the IFNy integrated association network (IAN) to identify novel regulatory mechanisms between IFNy signaling and PD-L1 expression. They also found that TGFB1 uniquely induces laminin pathway genes that explain the larger and more spread-out cells morphology of these cells in comparison to BMP2 induced cells. Overall, the results are interesting, the method is potentially of wide interest and the study is mostly well written. Experimental validation is a strength of their manuscript and lends confidence to their overall approach. However, there are some major statistical and data interpretation concerns that need to be addressed. Also, the manuscript would greatly benefit from additional case studies using a variety of datasets.<br><br>1. Statistical method validation is problematic or perhaps insufficiently explained. There is some data and discussion in Supplementary Figure 4 where the authors used shuffled (random) data to test their method. This analysis shows that even in random data, their method delivers hundreds to thousands of associations which indicates that the method is prone to false positive findings. This is a serious concern. Perhaps there is a way for the authors to derive association filtering cut-offs based on the values observed in shuffled data. They should also make show QQ plots to evaluate how their p-values of associations are distributed in true and random

data. Those would show whether the method is balanced or inflated.

***Nature Communications* and *Communications Biology* would both require you to address this concern about the statistical validation for further consideration.**

2. The authors only study one type of multi-omics data analysis scenario that is based on a) a single cell line MCF10A and b) contains several multi-omics datasets in a complex setup. As such, it is not clear whether a) the method is applicable to other kinds of molecular data, specifically patient -omics data that is likely much more heterogeneous, and b) whether all these different data modalities are required for the model to run successfully. Can MOBILE be applied to patient data, where columns are patients and rows are gene-level measurements? In the LOGO module, a patient would be left out or groups of patients, and gene association networks unique to that patient could be uncovered. Can the authors comment on the feasibility of extending MOBILE to integrate omics data across patients, rather than cell lines or ligand conditions? How about adding an example analysis that demonstrates the integration of just two data modalities, as a minimal case study ?

For further consideration with *Nature Communications* we would require you to add at least one additional dataset, ideally using patient data, to show feasibility. While *Communications Biology* would also encourage the inclusion of this kind of case study, at an absolute minimum the reliance on a single cell line should be further justified and clearly outlined as a limitation of the approach.

3. The authors need to illustrate how the ligand-specific association networks are obtained in more detail and possibly as a separate figure. On lines 208 to 210 as part of the figure 2 caption, the authors explain how the ligand-dependent coefficients are combined with coefficients that disappear from the FULL matrix to create a final ligand-specific associations list. These three lines are essential to understanding how ligand-specific IANs are generated, and should be emphasized.

This would be required for *Nature Communications* and *Communications Biology*.

4. In line with point 3, the caption for figure 2 is far too long and the figure contains too much information. I would suggest removing panel (e) since this is an overview of the two applications that are provided in more detail in figure 4 and 5. The method for identifying ligand-specific association networks should be explained in more detail in the text and may require its own figure altogether, which would mean panel (d) can be removed from figure 2 as well.

This would be required for *Nature Communications* and *Communications Biology*.

5. The authors obtain a “Robust Lasso coefficient matrix” by selecting the matrix with the highest number of coefficients that appears at least 5000 times in the 10000 lasso regression iterations. It would be informative to identify the variation across iterations of the algorithm and explore the gene associations that appear at

least 5000 times but are missed by the "Robust Lasso coefficient matrix". The last checkbox in Fig. 2b should be fixed, which says "Select the ensemble median", however in the methods it is described as a matrix with the highest number of coefficients that appear in at least 5000 iterations.

This would be required for *Nature Communications* and *Communications Biology*.

6. Does RPPA data include all proteins or only some proteins? If the coverage of RPPA is not proteome-wide, then it can induce major biases in their data integration because some or most proteins would lack signals. For example, how is this reflected in their network integration or GSEA? The latter analysis expects that all genes/proteins have some signal for ranking.

Please address this concern for *Nature Communications* and *Communications Biology*.

7. The validation of the identification of the association network seems to be suboptimal as they focus on the few top interactions. In addition to that, they should study if the statistical interactions they capture are significantly enriched in other previously-defined biochemical or genetic interaction networks (such as physical protein-protein interaction networks, genetic interactions, etc).

Please address this concern for *Nature Communications* and *Communications Biology*.

Minor suggestions:

• Line 47: "supervised learning (21–23), and machine learning (10,24)". Supervised learning is a form of machine learning
• Line 160 – "were" change to "are"
• Line 299 – "identified a five-gene set of connectors" to "identified five connectors"
• Line 555- "determine the coefficients depend on the" to "determine the coefficients that depend on the"
• Could not find the source data by searching for "doi:10.6084/m9.figshare.20294229."

The editors also cannot find the source data (doi.org/10.6084/m9.figshare.20294229)

• Some figures seem to miss panel labelling letters, such as Supplementary Figure 4.

Please address all minor concerns for all journals.

Reviewer #2 information

**Expertise**	Computational biology and machine learning; cellular pathways; integration of omics data
**Editor's comments**	This reviewer also finds the approach detailed here to be interesting, but has a number concerns, including the lack of citation and discussion of current approaches. They also agree that your manuscript would be improved with additional datasets.

Reviewer #2 comments

**Section**	**Annotated Reviewer Comments**
**Remarks to the Author: Overall significance**	This study proposed a new methodological framework for identifying context-specific networks and new regulatory mechanisms. The central dogma-based data integration is interesting. However, I have several concerns.<br><br>In the Introduction section, the authors say that there are currently two broad classes of approaches for data integration. In the field of bioinformatics, a variety of data integration methods have been developed (e.g., Kernel Matrices Integration, Canonical Correlation Analysis). Examples include the following papers:<br>● Brief Bioinform. 2018 Nov 29. doi: 10.1093/bib/bby115<br>● Bioinformatics. 2019 Jul 15;35(14):i474-i483. doi: 10.1093/bioinformatics/btz320<br>● Bioinformatics. 2018 Mar 15;34(6):1009-1015. doi: 10.1093/bioinformatics/btx682<br>● BMC Bioinformatics. 2021 Aug 4;22(1):392. doi: 10.1186/s12859-021-04303-4<br>● BMC Bioinformatics. 2013 Aug 12;14:245. doi: 10.1186/1471-2105-14-245<br>● BMC Bioinformatics. 2019 Jan 9;20(1):15. doi: 10.1186/s12859-018-2572-9<br>● Front Genet. 2021 Jul 22;12:607817. doi: 10.3389/fgene.2021.607817<br>● PLoS Comput Biol. 2021 Jun 1;17(6):e1009044. doi: 10.1371/journal.pcbi.1009044<br><br>A variety of supervised/semi-supervised/unsupervised network inference methods have been developed to identify biological pathways (e.g., Bayesian networks, Gaussian Graphical Models, Graphical Lasso, Pairwise Kernel). Examples include the following papers:<br>● Science. 2003 Oct 17;302(5644):449-53. doi: 10.1126/science.1087361

	• Biostatistics. 2018 Jan 1;19(1):71-86. doi: 10.1093/biostatistics/kxx017<br>• J Biomed Inform. 2012 Jun;45(3):535-43. doi: 10.1016/j.jbi.2012.02.004<br><br>The authors are supposed to discuss other related methods with the same goal. What are advantages of the proposed method over the other previous methods?<br>**For *Nature Communications* and *Communications Biology*, we would require you to have a more comprehensive literature comparison, including the papers mentioned here, and detail the novelty in your method over what we already have.**
**Remarks to the Author: Strength of the claims**	1. The authors insist that the proposed MOBILE method identify known biology and novel molecular associations. These findings in the Results section were not obtained by the other methods?<br>**For *Nature Communications* and *Communications Biology*, we ask that you detail the novel results obtained with your method and reflect this accurately in the text.**<br><br>2. Lasso is applied to the analysis of two datasets. Sparsity parameters of Lasso are optimized each time? How is the optimization is performed? What is the objective function? I'm afraid that the detected features with non-zero weights in the lasso model are heavily dependent on the sparsity parameter, which would affect the resulting biological interpretation.<br>**We would require you to address this concern for further consideration in *Nature Communications* and *Communications Biology*.**<br><br>3. The authors demonstrated the usefulness of the proposed method showing different case studies. Is there any consistency? What is the challenging biological problem behind the analyses in this study?<br>**Similarly to Reviewer #1, for further consideration in *Nature Communications*, we would require you to look at further case studies to show generalisability. As before, this point would be encouraged, but not required, for further consideration at *Communications Biology*.**<br><br>4. Why did the authors focus on the analysis of associations between IFNγ stimulation and PD-L1 regulation?<br>**For both point #4 and #5, please explain why you focused on these particular associations over others.**<br><br>5. Why did the authors focus on the analysis of BMP2 and TGFβ1n?<br><br>6. For the robust and parsimonious statistical associations between features of input data (line 148-157):

	It seems that the proposed method first calculates the robust parsimonious statistical association, and then iteratively uses the Lasso model for each dependent trait to measure the association with the independent variant analyte measure. It is not clear whether the authors confirmed the computational time of their proposed method.<br>**Please confirm the computational time for your method.**<br><br>7. For the multi-omics datasets from the LINCS consortium (line-500-501):<br>It is unclear why the authors included only 10% (RNAseq, ATACseq) and 20% (RPPA) highly variant analyses to evaluate their proposed method. The reasons should be clearly addressed.<br>**Please add in the reasoning here to your revised manuscript.**<br><br>8. What is the limitations of MOBILE? The authors should show limitations of the proposed method in addition to the advantages.<br>**Please add in the limitations of your method to your manuscript.**
**Remarks to the Author: Reproducibility**	The software is provided.

Open research evaluation

General information

Guidelines for Transparency and Openness Promotion (TOP) in Journal Policies and Practices ("TOP Guidelines")

The recommendations and requests in the table below are aimed at bringing your manuscript in line with common community standards as exemplified by the TOP Guidelines. While every publisher and journal will implement these guidelines differently, the recommendations below are all consistent with the policies at Nature Portfolio. In most cases, these will align with TOP Guidelines Level 2.

FAIR Principles

The goal of the recommendations in the table below related to **data or code** availability is to promote the FAIR Guiding Principles for scientific data management and stewardship (*Scientific Data* **3**: 160018, 2016). The FAIR Principles are a set of guidelines for improving 4 important aspects of digital research objects: **F**indability, **A**ccessibility, **I**nteroperability and **R**eusability.

ORCID

ORCID is a non-profit organization that provides researchers with a unique digital identifier. These identifiers can be used by editors, funding agencies, publishers, and institutions to reliably identify individuals in the same way that ISBNs and DOIs identify books and articles. Thus the risk of confusing your identity with another researcher with the same name is eliminated. **The ORCID website** provides researchers with a page where your comprehensive research activity can be stored.

Springer Nature collaborates with the ORCID organization to ensure that your research contributions (as authors and peer reviewers) are correctly attributed to you. Learn more at https://www.springernature.com/gp/researchers/orcid

Mandatory data deposition

Most scientific journals, including all Nature Portfolio journals, require that any newly-generated sequence data must be made publicly available before publication. There are some exceptions allowed for sensitive clinical data, but this should be discussed with the editor. All data must be deposited in a community-approved repository and accession codes/unique IDs must be included within the Data Availability Statement in the manuscript.

Examples of appropriate public repositories are listed below:

- GenBank (all DNA sequence data)
- Sequence Read Archive (high-throughput sequence data)
- Gene Expression Omnibus (Microarray or RNA sequencing data)

More information on mandatory data deposition policies at the Nature Portfolio can be found at http://www.nature.com/authors/policies/availability.html#data

Please visit this webpage for a list of approved repositories for various data types.

Data citation

Please cite (within the main reference list) any datasets stored in external repositories that are mentioned within their manuscript. For previously published datasets, we ask that you cite both the related research article(s) and the datasets themselves. For more information on how to cite datasets in submitted manuscripts, please see our data availability statements and data citations policy.

Citing and referencing data in publications supports reproducible research, by increasing the transparency and provenance tracking of data generated or analysed during research. Citing data formally in reference lists also helps facilitate the tracking of data reuse and may help assign credit for individuals' contributions to research. A number of Springer Nature imprints are signatories of the Joint Declaration on Data Citation Principles, which stress the importance of data resources in scientific communication.

Thank you for depositing your dataset in a public repository. In addition to providing the link within the Data Availability statement, we ask that you also cite the dataset in the main reference list.

Code availability and citation

Thank you for making your custom code available via Github. Upon publication, Nature Portfolio journals consider it best practice to release custom computer code in a way that allows readers to repeat the published results. Code should be deposited in a DOI-minting repository such as Zenodo, Gigantum or Code Ocean and cited in the reference list following the guidelines described in our policy pages (see link below). Authors are encouraged to manage subsequent code versions and to use a license approved by the open source initiative.

See here for more information about our code availability policies.

Ethics
We believe that authors, peer reviewers and editors should be required to disclose any competing interests that might influence their decisions and conclusions around a particular piece of content. In the interests of transparency and to help readers form their own judgements of potential bias, Nature Portfolio journals require authors to declare any competing financial and/or non-financial interests in relation to the work described.<br><br>Please provide a 'Competing interests' statement using one of the following standard sentences:<br>1. The authors declare the following competing interests: [specify competing interests]<br>2. The authors declare no competing interests.<br><br>See the Nature Portfolio competing interests policy for further information. The Springer Nature policy can be found here.
We believe that research that involves the use of clinical, biomedical or biometric data from human participants must only be carried out with the explicit consent of those whose data are involved. Consent must be obtained without any form of coercion and with participants' explicit understanding of the purpose for which their data will be used.<br><br>Because your study includes human participants, confirmation that all relevant ethical regulations were followed is needed for publication in any Springer Nature journal, and that informed consent was obtained. This must be stated in the Methods section, including the name of the board and institution that approved the study protocol.<br><br>Further details about the Nature Portfolio policy can be found at this webpage.
We believe that Springer Nature has a responsibility to support the relevant guidelines (based on research community or geographical region) that specify best practice in research and thus require all experimental results on animal and human participants to conform to the authors' local regulations and ethical standards, and we also encourage adherence to international standards.<br><br>Because your study uses live vertebrates, a statement affirming that you have complied with all relevant ethical regulations for animal testing and research is necessary. A statement explicitly confirming if the study received ethical approval, including the name of the board and institution that approved the study protocol is also required. The species, strain, sex and age of animals should be included.<br><br>Further details on our policies can be found at this webpage.
Cell line misidentification and cross-contamination is a common problem with serious consequences. Authors are asked to report on the source and authentication of their cell lines.
**Materials availability**

Oligo sequences, concentrations of antibodies, and sources of cell lines must be included in the Methods (these can also be provided in a main Table and cited in the Methods). Please see the Nature Portfolio policy page for further details:

Statistical reporting

Wherever statistics have been derived (e.g. error bars, box plots, statistical significance) figure legends should provide and define the n number (i.e. the sample size used to derive statistics) as a precise value (not a range), using the wording "n=X biologically independent samples/animals/cells/independent experiments/n= X cells examined over Y independent experiments" etc. as applicable. The figure legends must also indicate the statistical test used. Where appropriate, please indicate in the figure legends whether the statistical tests were one-sided or two-sided and whether adjustments were made for multiple comparisons. For null hypothesis testing, please indicate the test statistic (e.g. F, t, r) with confidence intervals, effect sizes, degrees of freedom and P values noted.

For examples of expected description of statistics in figure legends, please see the following:
https://www.nature.com/articles/s41467-019-11636-5 or
https://www.nature.com/articles/s41467-019-11510-4

When describing results as "significant" in the main text, please include details about the statistical test used and provide an exact p-value, rather than a significance threshold.

Please refer to these guidelines for detailed instructions about how your figures should be prepared. Following these instructions will reduce the chances of delays should we need to request replacement artwork from you at a later stage.

We strongly discourage deriving statistics from technical replicates, unless there is a clear scientific justification for why providing this information is important. Conflating technical and biological variability, e.g., by pooling technically replicates samples across independent experiments is strongly discouraged." **Please note that this information is missing in the legends of figures 4b; 5d.**

All error bars need to be defined in the legends (e.g. SD, SEM) together with a measure of centre (e.g. mean, median). For example, the legends should state something along the lines of "Data are presented as mean values +/- SEM" as appropriate. All box plots need to be defined in the legends in terms of minima, maxima, centre, bounds of box and whiskers and percentile. **Please note that the error bars need to be defined in the legend of figure 4b.**

The figure legends must indicate the statistical test used. Where appropriate, please indicate in the figure legends whether the statistical tests were one-sided or two-sided and whether adjustments were made for multiple comparisons. For null hypothesis testing, please indicate the test statistic (e.g. F, t, r) with confidence intervals, effect sizes, degrees of freedom and P

values noted. Please provide the test results (e.g. P values) as exact values whenever possible and with confidence intervals noted.

1. Please indicate the statistical test used for data analysis and where appropriate, please specify whether it was one-sided or two-sided and whether adjustments were made for multiple comparisons, in the legend of supplementary figure 2.
2. Please note that the exact p value should be provided, when possible, in the legend of supplementary figure 2.
3. Please note that for figure 3a, p-value is indicated in the legend; however, comparison for the same has not been represented in the figure. Please rectify this in the figure or legend, as applicable. Also, if appropriate, please provide the exact p-value and indicate the statistical test used for data analysis, specifying whether it was one-sided or two-sided and whether adjustments were made for multiple comparisons.

Please ensure that all micrographs include a scale bar and this scale bar is defined on the panels or in the figure legends. **Please note that the scale bar is missing for figure 5a.**

Figure legends/captions should not exceed 350 words. Please shorten by removing detailed methodological information and/or interpretation, or, if appropriate, consider splitting the affected figures in two.

Please make sure that the terms ‘atomic units (a. u.)’ or ‘arbitrary units (arb. units)’ are appropriately used.

Any abbreviations, symbols or colours present in your figures must be defined in the associated legends.
